# Adjunctive rifampicin for *Staphylococcus aureus* bacteraemia (ARREST): a multicentre, randomised, double-blind, placebo-controlled trial

**DOI:** 10.1016/S0140-6736(17)32456-X

**Published:** 2018-02-17

**Authors:** Guy E Thwaites, Matthew Scarborough, Alexander Szubert, Emmanuel Nsutebu, Robert Tilley, Julia Greig, Sarah A Wyllie, Peter Wilson, Cressida Auckland, Janet Cairns, Denise Ward, Pankaj Lal, Achyut Guleri, Neil Jenkins, Julian Sutton, Martin Wiselka, Gonzalez-Ruiz Armando, Clive Graham, Paul R Chadwick, Gavin Barlow, N Claire Gordon, Bernadette Young, Sarah Meisner, Paul McWhinney, David A Price, David Harvey, Deepa Nayar, Dakshika Jeyaratnam, Tim Planche, Jane Minton, Fleur Hudson, Susan Hopkins, John Williams, M Estee Török, Martin J Llewelyn, Jonathan D Edgeworth, A Sarah Walker, United Kingdom Clinical Infection Research Group (UKCIRG), Matthew Scarborough, Musa Kamfose, Ana de Veciana, Nicola Claire Gordon, Leon Peto, Gemma Pill, Tiphanie Clarke, Laura Watson, Bernadette Young, Dai Griffiths, Ali Vaughn, Luke Anson, Elian Liu, Sanuki Perera, Lydia Rylance-Knight, Carmen Cantell, Ruth Moroney, Jonathan D Edgeworth, Guy Thwaites, Karen Bisnauthsing, Antonio Querol-Rubiera, Charlotte Gibbs, Amita Patel, Carolyn Hemsley, Anna L Goodman, Duncan Wyncoll, Jason Biswas, Jennifer Fitzpatrick, Lizzie Roberts, James Millard, Neil Stone, Angela Cape, Lisa Hurley, Chi Kai Tam, Emmanuel Nsutebu, Marie-Claire Hoyle, Kate Maitland, Leona Trainor, Helen Reynolds, Jennifer Harrison, Jim Anson, Joseph Lewis, Jonathan Folb, Lynsey Goodwin, Nicholas Beeching, Sarah Dyas, Helen Winslow, Elizabeth Foote, Paul Roberts, Pavithra Natarajan, Alex Chrdle, Manuel Fenech, Hannah Allsop, Robert Tilley, Rachel Austin-Hutchison, Louise Barrett, Karen Brookes, Leanne Carwithen, Andrew Conbeer, Richard Cunningham, Charlotte Eglinton, Rosie Fok, Hannah Gott, Shona Hughes, Lewis Jones, Maggie Kalita, Angela King, Linda March, Mike Marner, Tracey Mynes, Aiden Plant, Suzanne Price, Judy Sercombe, Alison Stolton, Mark Wallis, Marie-Claire West, Jackie Westcott, Claire Williams, Rob Wosley, Leona Yabsley, Julia Greig, Laura Butland, Julie Sorrell, Tamara Mitchell, Abiola Alli, James Meiring, Boingotlo Masake, Carlene Rowson, Lynne Smart, Laura Makey, Sarah Moll, Jane Cunningham, Kim Ryalls, Kathryn Birchall, Janet Middle, Yvonne Jackson, Diane Swift, Joby Cole, Bala Subramanian, Faith Okhuoya, Maria Edwards, Cheryl Bailey, Rebecca Warren, Gayti Islam, Michael Ankcorn, Sarah Birchall, Paul Jones, John Humphries, Stephen Booth, Cariad Evan, Sarah Wyllie, Andrew Flatt, Lenka Strakova, Maria Hayes, Stacey Valentine, Clare James, Mary Wands, Nicolas Cortes, Nisa Khan, Robert Porter, Zoe Martin, Keith Yip, Helen Preedy, Helen Chesterfield, Tracey Dobson, Colin Walker, Martin Llewelyn, Angela Dunne, Laura Latter, Alison Porges, James Price, John Paul, Laura Behar, Louise Robinson, Amy Murray, Jennifer Fitzpatrick, Tenessa Sargent, Carrie Ridley, Laura Ortiz-Ruiz de Gordoa, Deborah Gilliam, Carole McPherson, Simon Matthews, Emma Foreman, Rajesh Jarghese, Alisha Beddoe, Sebastien Martin, Sephora Shaw, Dominika Wlazly, Maggie Cole, Abraham Gihawi, Kevin Cole, M Estée Török, Theodore Gouliouris, Luke Bedford, Rebecca B Saunderson, Ilias Mariolis, Rachel Bousfield, Isobel Ramsay, Daniel Greaves, Sani Aliyu, Kim Cox, Lois Mlemba, Lynne Whitehead, Naval Vyse, Mark Bolton, John Williams, Pauline Lambert, David Chadwick, Kirsty Baillie, Martyn Cain, Richard Bellamy, Jason Wong, Jane Thompson, Helen Vassallo, Agnieszka Skotnicka, Andrea Boyce, Anthony Donnelly, Peter Wilson, Graham FitzGerald, Victoria Dean, Kristian Warnes, Anna Reyes, Saadia Rahman, Lillian Tsang, Joanne Williams, Stephen Morris-Jones, Susan Hopkins, Elen Witness, Orla Brady, Elizabeth Woodford, Teresa Pettifer, Angela McCadden, Ben Marks, Sophie Collier, Damien Mack, Simon Warren, Colin Brown, Adrian Lyons, Sara Taiyari, Stephen Mepham, Anna Sweeney, Li-An Brown, Cressida Auckland, Alison Potter, Jess Mandiza, Maxine Hough, Sue Williams, Caroline Renton, Fiona Walters, Maria Nadolski, Andree Evans, Polly Tarrant, Katherine Curley, Sophie Whiteley, Julia Halpin, Melanie Hutchings, Shirley Todd, Christop Lohan, Tamika Chapter, Emma Folland, Alaric Colville, Katy Marden, Marina Morgan, Rosie Fok, Rob Porter, Mel Baxter, Jane Minton, Sarah Rippon, Muge Cevik, Judith Chapman, Tim Kemp, Rachel Vincent, Dave Osborne, Tracey Platt, James Calderwood, Bernadette Cook, Caroline Bedford, Leanne Galloway-Browne, Nadine Abberley, Kelly Attack, Joanna Allen, Pankaj Lal, Melanie Harrison, Sarah Stevenson, Carol Brooks, Paula Harlow, Jordan Ewing, Shirley Cooper, Roderick Balancio-Tolentino, Laura O'Neil, Rebecca Tagney, Daniela Shackcloth, Tim Planche, James Fellows, Ruth Millett, Jo Studham, Cherrelle de Souza, Geoffrey Howell, Hezron Greaves, Ella Foncel, Rahul Kurup, Jack Briggs, Melody Smith, Cristina Suarez, Giordana Sorrentino, Antonia Scobie, Angela Houston, Fozia Ahmad, Aodhan Breathnach, Rakhee Chahuan, Katie Wilkins, Achyut Guleri, Natalia Waddington, Rashmi Sharma, Peter Flegg, Veenu Kollipara, Mazhar Alam, Andrew Potter, Stacey Donaldson, Charlote Armer, Julie Frudd, Dakshika Jeyaratnam, Manju Joy, Asha Mathews, Stephen K Glass, Ayodele Ajayi, Amanda Fife, Saba Qaiser, Sharon Sheehan, Sergio Muñoz-Villaverde, Noah Yogo, Ines De Abreu, Gaynor Notcheva, Joanna Flanagan, Cordelia Watson, Efisia Sais, Adetunji Adedayo, Vicky Chu, Georgina Shaw, Michelle A Graver, Rebecca Palmer, Donna Palmer, Senait Haile, Joanne Gordon, Chi Kai Tam, Kirandip Mandar, Weronika Szypura, Neil Jenkins, Josephine Marange, Vusumuzi Shabangu, Katy Moore, Jill Lyons, Melinda Munang, Mirriam Sangombe, Ed Moran, Abid Hussain, Martin Wiselka, Adam Lewszuk, Sally Batham, Kate Ellis, Leila Bahadur, Helena White, Manish Pareek, Amandip Sahota, Stephen Coleman, Hilary Pateman, Atul Kotecha, Christopher Sim, Andrew Rosser, Jill Deane, Richard Nendick, Catherine Aldridge, Anne Clarke, Michelle Wood, Adele Marshall, Lynsey Stephenson, Tracy Matheson-Smith, John Sloss, Kathryn Potts, Joanne Malkin, Lemonia Ftika, Veena Raviprakash, Julian Sutton, Ahalya Malachira, Miranda Kean, Kristine Criste, Kirsty Gladas, Caroline Andrews, Clare Hutchison, Ellen Adams, Janet Andrews, Belinda Romans, Nicola Ridley, Melanie Ekani, Julie Mitchell, Nicola Smith, Tristan Clark, Sarah Glover, Robert Reed, Tat Yam, Holly Burton, Rasha Said, David Harvey, Amy Janvier, Reni Jacob, Chris Smalley, Alison Fair, Susan Lord, Kate Ripalda, Helen Wooldridge, Luis Cotter, Gus Cardoso, Elaine Strachan, Gagan Kaler, Adam Mohamoodally, Emma Lawrence, Zoe Prime, Rachel Abrahams, David Ashley Price, Lesley Rigden, Laura Shewan, Katherine Cullen, Ingrid Emmerson, Karen Martin, Hesther Wilson, Charley Higham, Kathryn Louise Taylor, Edmund Ong, Bijal Patel, Helena Bond, Janine Gradwell, John Widdrington, Clive Graham, Sarah Thornthwaite, Scott Prentice, Una Poultney, Hannah Crowther, Helen Fairlamb, Emily Hetherington, Chris Brewer, Suryabrata Banerjee, Clare Hamson, Anna McSkeane, Paul McWhinney, Paula Sharratt, Joanne Thorpe, Sue Kimachia, Helen Wilson, Benjamin Jeffs, Leslie Masters, Jonathan Wilson, Judith Platt, Lisa Burgess, Paul Chadwick, Adam Jeans, Claire Keatley, Amanda Moran, Zoe Swann, Katherine Pagett, Alex Peel, Jason Howard, Sarah Meisner, Kate Maloney, Avril Masdin, Louise Wright, Gavin Barlow, Samantha Crossman, Vicki Lowthorpe, Emma Moore, Peter Moss, Angela Parkin, Adam Wolstencroft, Bev Warner, Clare Tarbotton, Alison Eyre, Anne Anderson, Tina Burdett, Amy Driffill, Ann Sarah Walker, Fleur Hudson, Alex Szubert, Janet Cairns, Denise Ward, Helen Webb, Charlotte Russell, Brooke Jackson, Damilola Otiko, Chiara Borg, Lindsey Masters, Zaheer Islam, Carlos Díaz-Montaña, Debbie Johnson

**Affiliations:** a Nuffield Department of Medicine, University of Oxford, Oxford, UK; b Oxford University Clinical Research Unit, Ho Chi Minh City, Vietnam; c Medical Research Council Clinical Trials Unit, University College London, London, UK; d Royal Liverpool University Hospital, Liverpool, UK; e Plymouth Hospitals National Health Service (NHS) Trust, Plymouth, UK; f Sheffield Teaching Hospitals NHS Foundation Trust, Sheffield, UK; g Portsmouth Hospitals NHS Trust, Portsmouth, UK; h University College London Hospital National Health Service Foundation Trust, London, UK; i Royal Devon and Exeter NHS Foundation Trust, Exeter, UK; j Aintree University Hospital NHS Foundation Trust, Aintree, UK; k Blackpool Teaching Hospitals NHS Foundation Trust, Blackpool, UK; l Heart of England NHS Foundation Trust, Birmingham, UK; m University Hospital Southampton NHS Foundation Trust, Southampton, UK; n Department of Infection and Tropical Medicine, University Hospitals of Leicester NHS Trust, Leicester, UK; o Microbiology Department, Darent Valley Hospital, Dartford, UK; p North Cumbria University Hospitals NHS Trust, Carlisle, UK; q Salford Royal NHS Foundation Trust, Salford, UK; r Hull and East Yorkshire Hospitals NHS Trust, Hull, UK; s Royal United Hospitals Bath NHS Foundation Trust, Bath, UK; t Bradford Teaching Hospitals NHS Foundation Trust, Bradford, UK; u Newcastle upon Tyne Hospital NHS Foundation Trust, Newcastle, UK; v Wirral University Teaching Hospital NHS Foundation Trust, Birkenhead, UK; w County Durham and Darlington NHS Foundation Trust, Durham, UK; x King's College Hospital NHS Foundation Trust, London, UK; y St Georges University Hospitals NHS Foundation Trust, London, UK; z Leeds Teaching Hospitals NHS Trust, Leeds, UK; aa Royal Free London NHS Foundation Trust, London, UK; ab South Tees Hospitals NHS Foundation Trust, Middlesbrough, UK; ac University of Cambridge, Department of Medicine, Cambridge, UK; ad Brighton and Sussex Medical School, Brighton, UK; ae King's College London, London, UK

## Abstract

**Background:**

*Staphylococcus aureus* bacteraemia is a common cause of severe community-acquired and hospital-acquired infection worldwide. We tested the hypothesis that adjunctive rifampicin would reduce bacteriologically confirmed treatment failure or disease recurrence, or death, by enhancing early *S aureus* killing, sterilising infected foci and blood faster, and reducing risks of dissemination and metastatic infection.

**Methods:**

In this multicentre, randomised, double-blind, placebo-controlled trial, adults (≥18 years) with *S aureus* bacteraemia who had received ≤96 h of active antibiotic therapy were recruited from 29 UK hospitals. Patients were randomly assigned (1:1) via a computer-generated sequential randomisation list to receive 2 weeks of adjunctive rifampicin (600 mg or 900 mg per day according to weight, oral or intravenous) versus identical placebo, together with standard antibiotic therapy. Randomisation was stratified by centre. Patients, investigators, and those caring for the patients were masked to group allocation. The primary outcome was time to bacteriologically confirmed treatment failure or disease recurrence, or death (all-cause), from randomisation to 12 weeks, adjudicated by an independent review committee masked to the treatment. Analysis was intention to treat. This trial was registered, number ISRCTN37666216, and is closed to new participants.

**Findings:**

Between Dec 10, 2012, and Oct 25, 2016, 758 eligible participants were randomly assigned: 370 to rifampicin and 388 to placebo. 485 (64%) participants had community-acquired *S aureus* infections, and 132 (17%) had nosocomial *S aureus* infections. 47 (6%) had meticillin-resistant infections. 301 (40%) participants had an initial deep infection focus. Standard antibiotics were given for 29 (IQR 18–45) days; 619 (82%) participants received flucloxacillin. By week 12, 62 (17%) of participants who received rifampicin versus 71 (18%) who received placebo experienced treatment failure or disease recurrence, or died (absolute risk difference −1·4%, 95% CI −7·0 to 4·3; hazard ratio 0·96, 0·68–1·35, p=0·81). From randomisation to 12 weeks, no evidence of differences in serious (p=0·17) or grade 3–4 (p=0·36) adverse events were observed; however, 63 (17%) participants in the rifampicin group versus 39 (10%) in the placebo group had antibiotic or trial drug-modifying adverse events (p=0·004), and 24 (6%) versus six (2%) had drug interactions (p=0·0005).

**Interpretation:**

Adjunctive rifampicin provided no overall benefit over standard antibiotic therapy in adults with *S aureus* bacteraemia.

**Funding:**

UK National Institute for Health Research Health Technology Assessment.

## Introduction

*Staphylococcus aureus* bloodstream infection, also known as bacteraemia, is one of the most common and serious community-acquired and hospital-acquired bacterial infections worldwide.^1^ When *S aureus* enters the bloodstream it can disseminate to cause metastatic, deep-seated infection of almost any organ, with an associated mortality of approximately 20%.^2^ Despite the frequency and severity of *S aureus* bacteraemia, the optimal antibiotic treatment is uncertain. Fewer than 1600 participants have been enrolled in randomised trials of antibiotic therapy for the treatment of this infection over the past 50 years.^3^ Most treatment recommendations are therefore based on observational studies and clinical experience. Opinions on best management vary widely,^4^ but current guidelines^5^ recommend *S aureus* bacteraemia be treated with at least 14 days of an intravenous β-lactam antibiotic, or a glycopeptide if the bacteria are resistant to meticillin. Combination antibiotic therapy is generally not recommended, except in severe meticillin-resistant *S aureus* (MRSA) infections (eg, endocarditis and prosthetic joint infections); however, evidence in support of its use in such cases is weak.

**Research in context**

**Evidence before this study**

*Staphylococcus aureus* bacteraemia is probably the most common life-threatening, community-acquired and hospital-acquired infection worldwide, yet fewer than 1600 participants have been enrolled in randomised trials of antibiotic therapy for this infection over the past 50 years. For many years, the addition of rifampicin to an anti-staphylococcal penicillin or glycopeptide antibiotic has been hypothesised to improve outcomes from *S aureus* bacteraemia. A systematic review of relevant studies published before February, 2013, found three randomised trials and one cohort study, reporting a total of 98 participants with *S aureus* bacteraemia (54 rifampicin; 44 controls). A pooled analysis of data from these reports suggested rifampicin had no significant effect on all-cause mortality, but there was a trend for rifampicin to reduce clinical or bacteriologically proven treatment failure (odds ratio 0·38, 95% CI 0·13–1·11, p=0·08). No relevant randomised trials have been published since this systematic review. A 2017 post-hoc analysis of a prospective observational cohort of 964 patients with *S aureus* bacteraemia examined the effect of combination antibiotic therapy on patient outcome. Combination therapy was used in 53% of patients, with rifampicin used in 59% of combinations. Combination therapy was not associated with significant reductions in mortality overall, but 30-day and 60-day mortality and clinical complications were lower in a subgroup of patients with infected implanted foreign devices treated with combination therapy compared with patients without device-related infection. We searched PubMed up to July 1, 2017, for clinical trials with the terms “rifampicin” or “rifampin”, “*Staphylococcus aureu*s”, “bacteraemia”, and “bloodstream infection”.

**Added value of this study**

This trial is more than twice the size of the largest trial in *S aureus* bacteraemia to date, is a 50% increase in the total number of cases recruited in randomised trials of *S aureus* bacteraemia treatment over the past 50 years, and provides 95% CIs around our estimates of no difference between rifampicin and placebo that lie within 7·5%—smaller than the 10% non-inferiority margins recommended by licensing authorities for antibiotic trials. Although designed to test the superiority of rifampicin, the trial provides convincing evidence of non-inferiority of rifampicin to placebo—ie, convincing evidence of absence of benefit.

**Implications of all the available evidence**

Although adjunctive rifampicin does not reduce mortality from *S aureus* bacteraemia, it might reduce the risk of disease recurrence, but our results suggest this effect had no impact on short-term or long-term mortality. Furthermore, rifampicin complicates other drug treatment. We consider that adjunctive rifampicin provides no overall benefit over standard antibiotic therapy in adults with *S aureus* bacteraemia.

Adjunctive rifampicin has long been hypothesised to improve outcomes for serious *S aureus* infections.^6^ It has good oral bioavailability and penetrates cells, tissues, and biofilms better than β lactams and glycopeptides; therefore, in combination with these agents, rifampicin might eradicate serious *S aureus* infections more effectively than either drug alone.^7^ The use of adjunctive rifampicin in the treatment of *S aureus* bacteraemia varies widely worldwide, although case-series from the UK^8^ and Germany^9^ reported nearly a third (86 of 274 and 301 of 964) of all adults with *S aureus* bacteraemia received rifampicin, particularly patients with deep-seated infections. However, evidence to support benefit of treatment is weak, with rifampicin associated with hepatic toxicity and substantial interactions with other drugs.^8^, ^9^, ^10^ A systematic review^11^ identified four studies (three randomised trials and one cohort study) that included 54 participants with *S aureus* bacteraemia treated with adjunctive rifampicin and 44 standard-therapy controls, and showed rifampicin to be associated with reduced all-cause mortality and clinical or bacteriological failure; although data were too few to make definitive treatment recommendations.

Given the substantial mortality associated with *S aureus* bacteraemia, the widespread use of adjunctive rifampicin in its treatment, and uncertainty whether rifampicin's benefits outweigh its risks, a large pragmatic trial was needed. In ARREST, we tested the hypothesis that adjunctive rifampicin reduces bacteriologically confirmed treatment failure or disease recurrence, or death by enhancing early killing of *S aureus*, sterilising infected foci and blood faster, thereby reducing the risk of dissemination and metastatic infection.^12^

## Methods

### Study design and participants

We did a multicentre, randomised, double-blind, placebo-controlled trial of adjunctive rifampicin in adults with *S aureus* bacteraemia treated in 29 UK hospitals. The trial was approved by the London (Westminster) Research Ethics Committee (12/LO/0637). Adult inpatients (≥18 years) were eligible if they had: symptoms and signs of *S aureus* infection; meticillin-susceptible *S aureus* (MSSA), or MRSA, grown from at least one blood culture; received ≤96 h of active antibiotic therapy for the current infection (allowing any stat [one-off] doses >96 h earlier); no pre-existing evidence of *S aureus* rifampicin non-susceptibility; and no contraindications to rifampicin. Participants were ineligible if *S aureus* was considered a blood culture contaminant or mixed with another organism likely to be contributing to the current infection, active tuberculosis was suspected, rifampicin was considered mandatory for any reason, or the subject had previously been randomised in ARREST. Participants, or their legal representatives (in the case of incapacity), gave written informed consent. Patients were recruited by the study team and in consultation with the hospital team responsible for the patient's in-hospital care.

### Randomisation and masking

Participants were randomly assigned (1:1) to receive either rifampicin or placebo for 2 weeks, plus standard backbone antibiotic therapy as chosen by the attending physician. Randomisation was stratified by centre. A computer-generated sequential randomisation list using variably sized permuted blocks was prepared by the trial statistician, and incorporated securely into the online trial database. The list was concealed until allocation by the prevention of access by any database users through login-based permissions; after eligibility was confirmed, researchers at the local hospitals then did the randomisation.

Trial doctors and nurses, and other nurses (except any ward nurses reconstituting intravenous trial drug) and doctors who cared for the participants during normal clinical care, were masked to the group allocations and treatment; hospital pharmacists and trial statisticians were unmasked. Trial centres were supplied with individual participant blinded treatment packs, which were labelled only with a trial number, and contained either active rifampicin (300-mg capsules) or identical placebo capsules sufficient for 14 days of treatment at 900 mg, according to the randomisation list. Rifampicin capsules were over-encapsulated to make them indistinguishable from placebo. For participants requiring intravenous treatment, hospital pharmacists dispensed rifampicin for infusion or saline from local pharmacy stock, with an opaque cover to mask the treatment.

Rifampicin can turn urine (and tears and sweat) reddish-orange—an effect impossible to safely replicate with a placebo. Therefore, urine discolouration might have been a potential source of treatment unmasking, particularly of the participant; however, this effect is variable both within and between individuals. The opportunity for physicians to examine the urine at the bedside only occurred in participants with urinary catheters. Catheters were not required by all participants and were removed at the earliest opportunity. The success of blinding was assessed at the final 12-week visit, at which time physicians and participants were asked which treatment they believed they had received.

### Procedures

As decided by the attending physician, 600 mg or 900 mg of rifampicin was given per day according to weight, either as a divided dose given twice per day or a single dose given once per day, orally or intravenously for 14 days or until cessation of backbone antibiotic treatment for the bacteraemia—whichever occurred first. Information on all antibiotics received from randomisation to 12 weeks was collected, but not according to specific indication. Consultation with an infection specialist, with advice on management to non-specialists caring for the trial participants, followed normal clinical practice at all sites. Attending physicians could change backbone antibiotics according to clinical need and advice of the infection specialist, and could use open-label rifampicin after 14 days. In cases judged clinically necessary, participants could stop the masked trial drug before the end of the 2-week period to use open-label rifampicin, with participants continuing follow-up off study drug, on study. Primary antibiotic treatment and duration was defined by complete cessation of all antibiotics for 2 days, with the exception of vancomycin, in which case intermittent dosing up to 1 week was allowed. The cessation of vancomycin was defined by adding the number of days between the last two doses to the date of the final dose.

Participants left the trial after 12 weeks, with clinical assessments in hospital on days 0, 3, 7, 10, and 14, and then once per week until either discharge or week 12—whichever occurred first. Blood cultures were done on days 0, 3, and 7; C-reactive protein concentration was measured on days 3, 7, 10, and 14; and liver function tests were done on days 3 and 10. Ideally, the final visit at week 12 was done face-to-face, but could also be over the telephone or via general practitioner records; this visit occurred any time after week 11. Consent was obtained to confirm vital status on all participants at trial closure.

### Outcomes

The primary outcome in the final protocol was time to bacteriologically confirmed treatment failure or disease recurrence, or death (all-cause), from randomisation to 12 weeks. Bacteriologically confirmed treatment failure was defined as symptoms and signs of infection ongoing for more than 14 days from randomisation, with *S aureus* isolated from blood or another sterile site (eg, joint fluid and pus from tissue). Bacteriologically confirmed disease recurrence was *S aureus* isolated from a sterile site after more than 7 days of apparent clinical improvement. As defined, treatment failure reflected both the speed of killing of *S aureus* and sterilisation of infected foci and blood, and both treatment failure and disease recurrence reflected the risk of dissemination and metastatic infection. Secondary outcomes were time to all-cause mortality from randomisation to 2 weeks; time to death or clinically defined treatment failure or disease recurrence from randomisation to 12 weeks; duration of bacteraemia (to assess whether rifampicin was associated with faster killing of *S aureus* and sterilisation of infected foci and blood); grade 3–4 adverse events (AEs; graded following the Common Toxicity Criteria for AEs 4.03^13^), serious adverse events (SAEs), antibiotic or trial-drug modifying AEs (all AEs reported, primary comparisons based on time to first event); the proportion for whom treatment was modified (including concomitant medications) because of drug interactions; and the proportion who developed rifampicin-resistant *S aureus* (from sterile sites). All potential treatment failures or disease recurrences and deaths were adjudicated by an independent review committee masked to the treatment.

### Statistical analysis

The trial was originally designed with two co-primary outcomes: all-cause mortality by 14 days and bacteriologically confirmed treatment failure or disease recurrence, or death, by week 12. Assuming 80% power, a two-sided α of 0·025 (to adjust for multiple testing given two co-primary outcomes), and a 10% loss to follow-up by week 12, 920 participants were needed to detect a 30% relative reduction in treatment failure or disease recurrence, or death, from 35% to 25%—an absolute difference of 10%, corresponding to a number needed to treat of ten. Assuming 80% power, a two-sided α of 0·025, and a 4% loss to follow-up by 14 days (as most participants remained in hospital during this time), 940 participants were needed to detect a 45% relative reduction in mortality from 16% to 9%—an absolute difference of 7%, corresponding to a number-needed-to-treat of 14. Therefore, the total sample size was originally 940 participants.

Recruitment to the trial was slower than anticipated. To facilitate successful completion of the trial, and at the request of the trial funder, after 3 years of recruitment the 14-day mortality was changed from a co-primary to a secondary outcome. 12-week bacteriologically confirmed treatment failure or disease recurrence, or death, therefore became the sole primary outcome, with a consequent decrease in sample size (due to increase in the two-sided α [type I error] from 0·025 [two co-primary outcomes] to 0·05 [one primary outcome]). With 12-week bacteriologically confirmed treatment failure or disease recurrence, or death, as the sole primary outcome, the total sample size became 770 participants (α=0·05, other assumptions as above).

Interim data were reviewed by an independent data monitoring committee (four annual meetings) using Haybittle-Peto criterion (p<0·001). Randomised groups were compared following the principle of intention-to-treat (including all follow-up regardless of changes to treatment) using log-rank tests for time-to-event outcomes, exact tests for binary outcomes, and generalised estimating equations with independent working correlation for global tests of repeated measures. Primary analyses were not stratified by centre. A deep infection focus was defined as infection of implanted vascular device, native or prosthetic heart value, native or prosthetic bone or joint, or deep tissue infection or abscess (including vertebral bone or disc or other bone infection, epidural or intraspinal empyema, infected intravascular thrombus and brain infection). Additional details are provided in the appendix pp 4–7. Analyses were done with the use of Stata version 14.2.

The trial was registered, number ISRCTN37666216.

### Role of the funding source

The funder had no role in the study design (other than requesting a change from two co-primary outcomes to one primary outcome because of slow recruitment, as described earlier); in the collection, analysis, and interpretation of data; in the writing of the report; and in the decision to submit the paper for publication. AS and ASW had complete access to the data. GET was responsible for the decision to submit the manuscript for publication.

## Results

**Figure 1:**
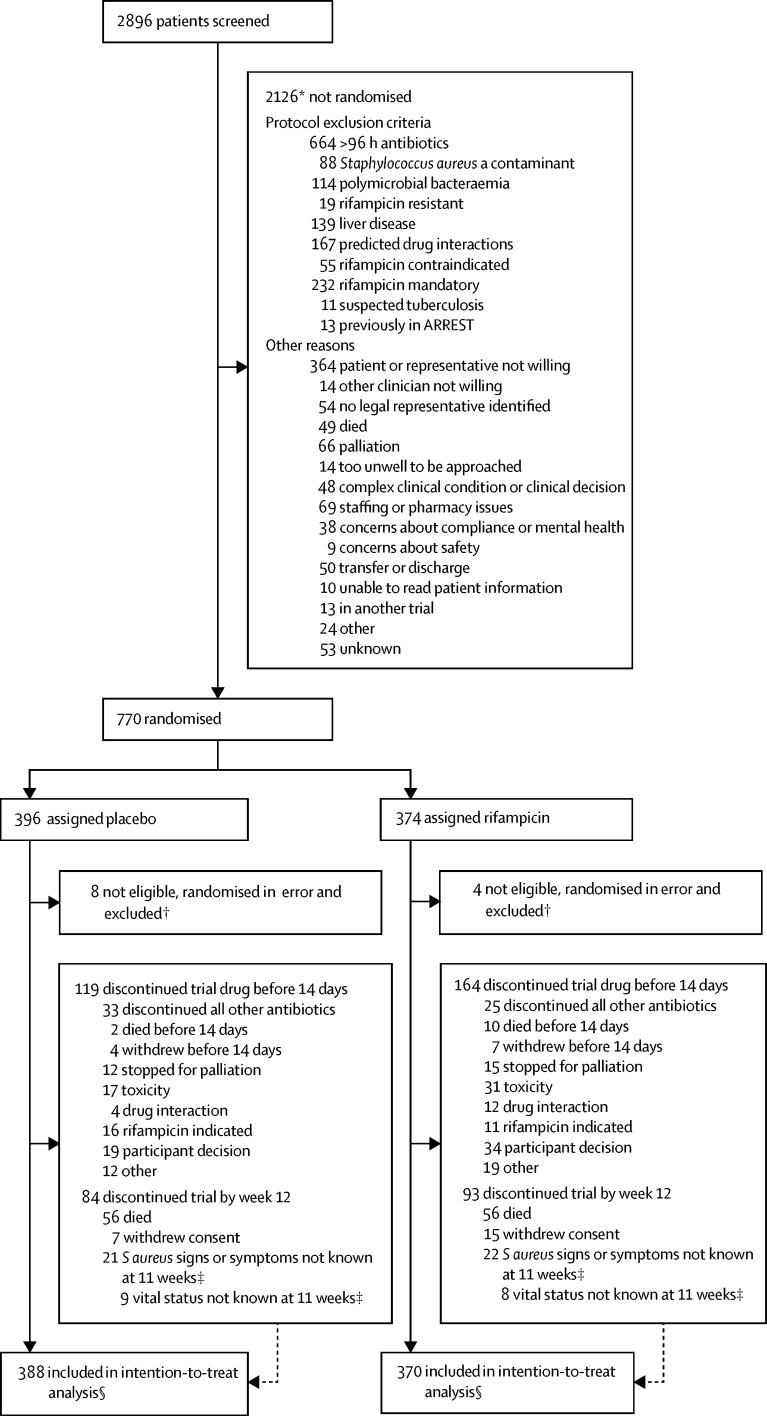
Trial profile *Reasons are not mutually exclusive; therefore, total is more than the number of participants not randomly assigned. †Seven participants with predicted drug interaction, two misdiagnosed (*S aureus* not grown from blood but only other samples), rifampicin considered mandatory in one, one other clinician considered participant should not have been assigned because of acute kidney injury, one other clinician considered participant should not have been randomly assigned because they were in another study (not of an investigational medicinal product). ‡Final 12-week visit could occur any time from 11 weeks onwards according to the protocol. Consent withdrawals not included in these numbers. §Time-to-event analyses included all time at risk from randomisation to the earliest of the event or last clinical follow-up if the event had not occurred.

Between Dec 10, 2012, and Oct 25, 2016, 770 participants from 29 UK hospital groups were randomly assigned to add placebo (n=396) or rifampicin (n=374) to their backbone antibiotic treatment (figure 1). 12 participants were randomly assigned (eight to placebo and four to rifampicin) in error (ie, the participant should not have been assigned and never received trial drug) and were excluded, leaving 758 (388 placebo and 370 rifampicin) participants in the analyses.

Baseline characteristics were similar between randomised groups (table 1; appendix p 14). 495 (65%) were men, with a median age of 65 (IQR 50–76) years, and a Charlson comorbidity score of 2 (0–3). 70 (9%) participants were in an intensive care unit. Mean C-reactive protein was 164 (SE 3·7) mg/L. 127 (17%) participants had consent provided by a legal representative because of incapacity. 485 (64%) *S aureus* infections were community acquired, 132 (17%) were nosocomial, and 47 (6%) were caused by MRSA. No patients were known to have rifampicin-resistant *S aureus* bacteraemia at randomisation. The initial focus was deep in 301 (40%) participants, including 33 (4%) with endocarditis and 14 (2%) with infected prostheses; 130 (17%) infections were due to infected central or peripheral lines, or both; 138 (18%) infections were associated with skin or soft tissue infections, or both; another type of focus was identified in 49 (6%) participants and not established in 139 (18%) participants. At randomisation, the median time for which participants had received active antibiotics was 62 (IQR 42–75) h.

**Table 1:** Characteristics at randomisation

		**Placebo (n=388)**	Rifampicin (n=370*)	Total (N=758*)
Men		246 (63%)	249 (67%)	495 (65%)
Age at last birthday (years)		66 (51–76)	64 (49–76)	65 (50–76)
Mode of acquisition of infection*				
	Community acquired	240 (62%)	245 (66%)	485 (64%)
	Nosocomial infection (onset ≥48 h after admission)	76 (20%)	56 (15%)	132 (17%)
	Health-care associated (all other)	72 (19%)	68 (18%)	140 (18%)
Meticillin-resistant *Staphylococcus aureus*		21 (5%)	26 (7%)	47 (6%)
Main focus or foci of infection*†				
	Native heart valve	16 (4%)	17 (5%)	33 (4%)
	Native joint	34 (9%)	29 (8%)	63 (8%)
	Prosthetic heart valve or joint‡	5 (1%)	9 (2%)	14 (2%)
	Implanted vascular device (other than intravenous catheter)	23 (6%)	13 (4%)	36 (5%)
	Deep tissue infection or abscess	94 (24%)	82 (22%)	176 (23%)
	Central or peripheral intravenous catheter	67 (17%)	63 (17%)	130 (17%)
	Skin or soft tissue (excluding wounds)	66 (17%)	72 (19%)	138 (18%)
	Surgical wound	15 (4%)	10 (3%)	25 (3%)
	Pneumonia or urinary tract infection	30 (8%)	30 (8%)	60 (8%)
	Not established	67 (17%)	72 (19%)	139 (18%)
Any deep-seated focus§		159 (41%)	142 (38%)	301 (40%)
Admitted to intensive care unit*		36 (9%)	34 (9%)	70 (9%)
C-reactive protein (mg/L; n=755)¶		163 (5·2)	166 (5·3)	164 (3·7)
SOFA score*		2 (1–4)	2 (1–4)	2 (1–4)
Charlson comorbidity score*		2 (0–3)	1 (0–3)	2 (0–3)
	Cancer (n=756)	60 (16%)	66 (18%)	126 (17%)
	Chronic lung disease (n=756)	42 (11%)	48 (13%)	90 (12%)
	Moderate or severe renal disease (n=755)	80 (21%)	58 (16%)	138 (18%)
	Diabetes*	119 (31%)	109 (30%)	228 (30%)
Active injecting drug use (n=751)		41 (11%)	42 (11%)	83 (11%)
Days between drawing of first positive blood culture and randomisation*		3 (2–3)	3 (2–4)	3 (2–3)
Hours of active antibiotic therapy before randomisation		63 (42–75)	60 (41–76)	62 (42–75)
Rifampicin-resistant infection at randomisation (n=750)||		0	0	0

Data are n (%), median (IQR), or mean (SE). As an indicator of imbalance, p>0·05 for all comparisons of baseline characteristics between groups. SOFA=sequential organ failure assessment.

* One rifampicin participant withdrew shortly after randomisation without having completed an enrolment form: most baseline characteristics are therefore missing for this participant. If any other participants had missing data, then denominators are shown.

† Individuals could have multiple foci, so sum is more than total randomised.

‡ Two placebo, five rifampicin with prosthetic heart valves; three placebo, four rifampicin with prosthetic joints.

§ Infection of implanted vascular device, native or prosthetic heart valve, native or prosthetic bone or joint, deep tissue infection or abscess (including vertebral bone or disc or other bone infection, epidural or intraspinal empyema, infected intravascular thrombus, brain infection).

¶ Mean (SE) estimated using normal interval regression to account for values above limit of quantification in one centre (see appendix p 5).

|| Not required to be known at the point of randomisation for eligibility.

744 (98%) participants initiated blinded trial drug a median of 68 (IQR 48–85) h after starting active antibiotics for the current infection (table 2). Participants continued treatment with the trial drug for a median of 12·6 (IQR 6·0–13·2) days in the rifampicin group versus 13·0 (IQR 11·3–13·5) days in the placebo group (p<0·0001; primarily due to antibiotic-modifying AEs and drug–drug interactions, discussed later). The proportion of participants who reported missing any doses ranged from 18 (9%) of 190 to 58 (16%) of 357 but did not differ between randomised groups (global p=0·72; appendix p 8).

**Table 2:** Trial drug and backbone antibiotic treatment

			**Placebo (n=388)**	**Rifampicin (n=370)**	**Total (N=758)**
Trial drug					
	Never initiated trial drug		8 (2%)	6 (2%)	14 (2%)
	Initiated intravenous trial drug		51 (13%)	45 (12%)	96 (13%)
	Initiated oral trial drug		329 (85%)	319 (86%)	648 (85%)
	Initiated trial drug once per day		175 (45%)	173 (47%)	348 (46%)
	Initiated trial drug twice per day		205 (53%)	191 (52%)	396 (52%)
	Initiated trial drug 600 mg per day		74 (19%)	75 (20%)	149 (20%)
	Initiated trial drug 900 mg per day		306 (79%)	289 (78%)	595 (78%)
	Initial total dose per day (mg/kg; n=741)		11·2 (9·9–12·9)	11·0 (10·0–12·7)	11·1 (10·0–12·9)
Hours from starting active antibiotics to trial drug			69 (49–85)	68 (46–85)	68 (48–85)
Days on trial drug			13·0 (11·3–13·5)	12·6 (6·0–13·2)	12·8 (7·9–13·4)
Backbone active antibiotic treatment*					
	Flucloxacillin		321 (83%)	298 (81%)	619 (82%)
	Co-amoxiclavulante		122 (31%)	107 (29%)	229 (30%)
	Piperacillin or tazobactam		115 (30%)	102 (28%)	217 (29%)
	Vancomycin or teicoplanin		188 (48%)	192 (52%)	380 (50%)
	Cephalosporin		110 (28%)	104 (28%)	214 (28%)
	Fluoroquinolone		47 (12%)	46 (12%)	93 (12%)
	Macrolide		30 (8%)	28 (8%)	58 (8%)
	Clindamycin		23 (6%)	36 (10%)	59 (8%)
	Tetracycline		29 (7%)	26 (7%)	55 (7%)
	Gentamicin or amikacin		101 (26%)	98 (26%)	199 (26%)
		Stat gentamicin or amikacin	95 (24%)	87 (24%)	182 (24%)
	Carbapenem		38 (10%)	35 (9%)	73 (10%)
	Other antibiotic†		52 (13%)	52 (14%)	104 (14%)
	Number of antibiotics received during *Staphylococcus aureus* infection episode (excluding study drug)		3 (2–4)	3 (2–4)	3 (2–4)
Days of antibiotic treatment for *S aureus* infection episode			30 (18–44)	29 (17–45)	29 (18–45)
Rifampicin used open-label: initiated <14 days from randomisation‡			25 (6%)	18 (5%)	43 (6%)
Rifampicin used open-label: initiated ≥14 days from randomisation			27 (7%)	14 (4%)	41 (5%)

Data are n (%), or median (IQR).

* Including active antibiotics taken from the first blood culture sample throughout the illness episode.

† Open-label rifampicin excluded.

‡ Masked trial drug stopped and open-label rifampicin initiated for clinical reasons.

Various backbone active antibiotics were used (table 2); however, flucloxacillin was given to 619 (82%) participants, and vancomycin or teicoplanin to 380 (50%) at some point in the primary treatment course. The median number of antibiotics used (3 [IQR 2–4]) and the duration of anti-staphylococcal treatment (29 [IQR 18–45] days) was similar between groups (table 2). 32 (9%) participants in the rifampicin group versus 52 (13%) in the placebo group used open-label rifampicin (p=0·04), initiated a median of 14 (IQR 7–18) days after randomisation (table 2; appendix p 15). 159 participants who received placebo versus 142 who received rifampicin had a deep focus, which was drained or removed in 35 (22%) versus 29 (20%), a median of 5 (IQR 2–12) days and 3 (IQR 1–6) days after randomisation (appendix p 16).

22 (3%) participants withdrew consent. At the 12-week visit, only 39 (5%) participants had unknown vital status and 65 (9%) were not assessed for signs or symptoms of *S aureus* infection (including consent withdrawals). 23 (3%) participants were still in hospital at week 12; the median duration of initial hospital admission was 21 (IQR 14–50) days in the placebo group versus 22 (13–43) days in the rifampicin group (p=0·80). 94 (24%) participants in the placebo group versus 83 (22%) in the rifampicin group were re-admitted after discharge and before week 12.

**Figure 2:**
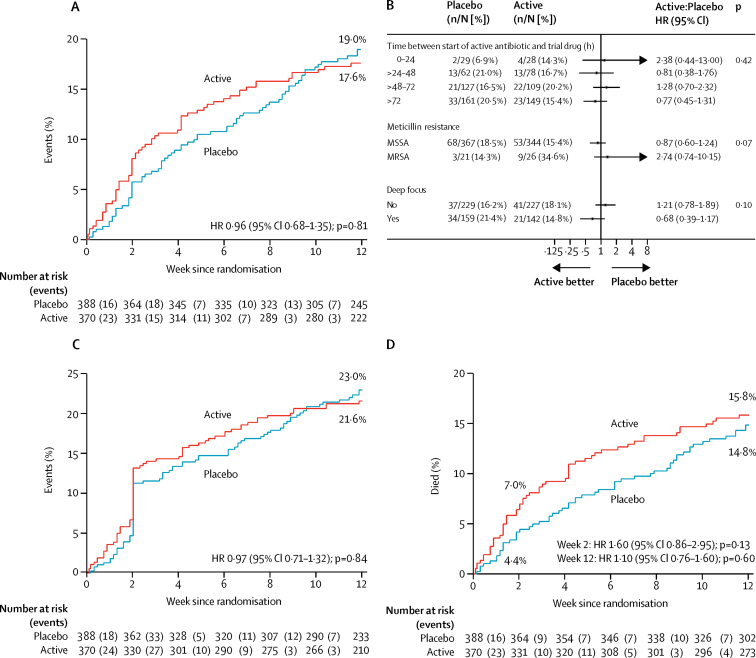
Treatment failure, disease recurrence, and death from randomisation to 12 weeks Kaplan-Meier curves of bacteriologically confirmed treatment failure or disease recurrence, or death, (A), clinically defined treatment failure or disease recurrence, or death, (C), and mortality (D) from randomisation to 12 weeks; and Forest plot of three priority subgroup analyses for bacteriological treatment failure or disease recurrence, or death from randomisation to 12 weeks (primary endpoint; B). The p value for interaction is presented in (B). HR=hazard ratio. MSSA=meticillin-susceptible *Staphylococcus aureus*. MRSA=meticillin-resistant *S aureus*.

By week 12, bacteriologically confirmed treatment failure or disease recurrence, or death, had occurred in 62 (17%) participants in the rifampicin group versus 71 (18%) in the placebo group (absolute risk difference [RD] −1·4%, 95% CI −7·0 to 4·3; hazard ratio [HR] 0·96, 0·68–1·35, p=0·81; figure 2; per protocol analysis, appendix p 7). In exploratory post-hoc analyses comparing rifampicin with placebo (table 3), four (1%) versus five (1%) treatment failures (competing risks p=0·82), three (1%) versus 16 (4%) disease recurrences (competing risks p=0·01), and 55 (15%) versus 50 (13%) deaths without bacteriological treatment failure or disease recurrence (competing risks p=0·30) were reported. The number needed to treat to prevent one bacteriologically confirmed recurrence was 29.

**Table 3:** Failure, recurrence, and causes

			**Bacteriological failure or recurrence**			**Clinical failure or recurrence**			**Deaths (all)**	
			Placebo	Rifampicin	p value	Placebo	Rifampicin	p value	Placebo	Rifampicin
Total randomised			388	370	··	388	370	··	388	370
Total events			71 (18%)	62 (17%)	0·81	86 (22%)	76 (21%)	0·84	56 (14%)	56 (15%)
Failure			5 (1%)	4 (1%)	0·82	25 (6%)	23 (6%)	0·97	··	··
	Failure due to slow resolution		3 (1%)	1 (<1%)	··	17 (4%)	10 (3%)	··	··	··
Recurrence			16 (4%)	3 (1%)	0·01	23 (6%)	8 (2%)	0·01	··	··
Death without either			50 (13%)	55 (15%)	0·30	38 (10%)	45 (12%)	0·22	··	··
Total failures or recurrences, or *Staphylococcus aureus*-related deaths attributed by Endpoint Review Committee			21 (100%)	7 (100%)	··	48 (100%)	31 (100%)	··	32 (100%)	36 (100%)
	Failure of antibiotics		1 (5%)	0	··	3 (6%)	1 (3%)	··	1 (3%)	3 (8%)
	Failure of source management		17 (81%)	3 (43%)	··	38 (79%)	24 (77%)	··	21 (66%)	18 (50%)
		Not recognised	9 (43%)	2 (29%)	··	12 (25%)	5 (16%)	··	3 (9%)	4 (11%)
		Recognised, not actively managed	5 (24%)	1 (14%)	··	16 (33%)	14 (45%)	··	8 (25%)	8 (22%)
		Recognised, actively managed, still failed or recurred	3 (14%)	0	··	10 (21%)	5 (16%)	··	10 (31%)	6 (17%)
	Not possible to distinguish		3 (14%)	4 (57%)	··	7 (15%)	6 (19%)	··	10 (31%)	15 (42%)
	Death a consequence of late presentation		··	··	··	··	··	··	3 (9%)	11 (31%)

Data are n (%). Percentages in the top half of the table are the proportion of total randomised patients, and in the bottom half are the proportion of total failures or recurrences, or *S aureus*-related deaths.

Of 28 failures or recurrences in which *S aureus* was isolated from a sterile site, paired baseline and failure or recurrence isolates were stored for 11 (39%). All failure and recurrence isolates were whole-genome sequenced and within 12 single nucleotide variants of the baseline isolate (median 1 [IQR 1–6], range 0–12).

Subgroup analyses according to time between starting active antibiotics and trial drug, meticillin resistance, and foci of infection (deep *vs* not deep), suggested no heterogeneity in absence of effect of rifampicin (p_heterogeneity_=0·42, 0·07, 0·10; figure 2). The effect of rifampicin varied significantly, according to the initial antibiotic given at randomisation, with some suggestion of benefit in those with meticillin-sensitive infection treated with flucloxacillin alone (p_heterogeneity_=0·01, appendix p 9), but across none of 16 other subgroup analyses (p_heterogeneity_>0·05, appendix pp 9, 10).

Clinically defined treatment failure or disease recurrence, or death, occurred in 76 (21%) participants in the rifampicin group versus 86 (22%) in the placebo group (RD −1·4%, 95% CI −7·4 to 4·7; HR 0·97, 0·71–1·32, p=0·84; figure 2). In exploratory post-hoc analyses comparing the rifampicin and placebo groups, 23 (6%) versus 25 (6%) failures (competing risks p=0·97), eight (2%) versus 23 (6%) recurrences (competing risks p=0·01), and 45 (12%) versus 38 (10%) deaths without clinically defined treatment failure or disease recurrence (competing risks p=0·22) were reported (table 3). The number needed to treat to prevent one clinically confirmed disease recurrence was 26. The endpoint review committee adjudicated that failure of infection focus management was implicated in 24 (77%) of 31 failures or recurrences on rifampicin versus 38 (79%) of 48 on placebo; including five (16%) versus 12 (25%) in which the focus was not recognised (table 3). 34 (9%) of 370 participants in the rifampicin group received antibiotics after the primary treatment course, versus 60 (15%) of 388 in the placebo group (p=0·01).

**Figure 3:**
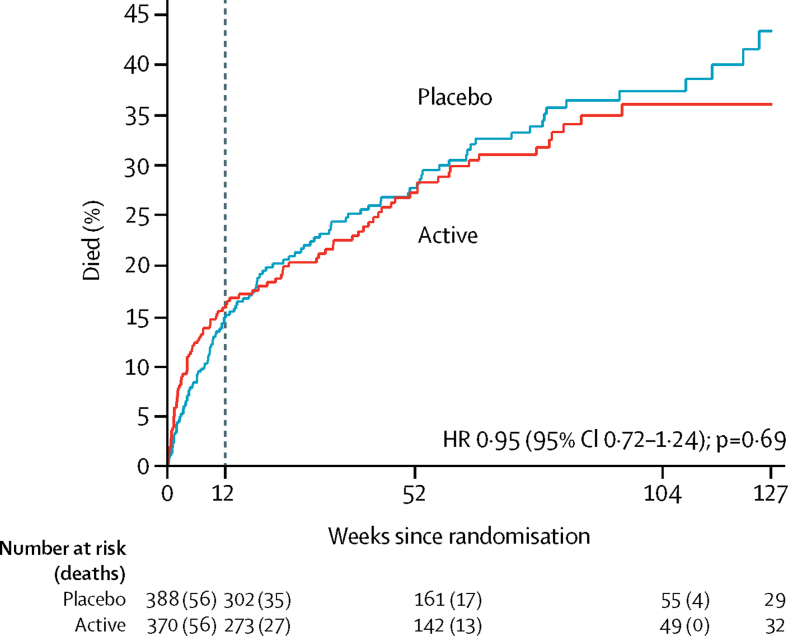
Long-term mortality Dashed line indicates end of formal trial follow-up. HR=hazard ratio.

By week 12, 56 (15%) participants in the rifampicin group versus 56 (14%) in the placebo group had died (RD 1·0%, 95% CI −4·3 to 6·2; HR 1·10, 0·76–1·60; p=0·60; figure 2). 25 (7%) participants in the rifampicin group versus 17 (4%) in the placebo group died by week 2 (HR 1·60, 95% CI 0·86–2·95, p=0·13). Of the reported deaths, 14 in the rifampicin group versus 16 in the placebo group were definitely *S aureus*-related, 14 versus 12 were probably *S aureus*-related, and eight versus four were possibly *S aureus*-related (appendix p 17). 18 deaths in the rifampicin group versus 23 in the placebo group were not attributed to *S aureus* (remainder unattributable; overall p=0·64). There was no difference in long-term (post-week 12) survival between the groups (p=0·69; figure 3).

There was no evidence that duration of bacteraemia was significantly shorter in those randomly assigned to rifampicin (global p=0·66; appendix p 11). C-reactive protein concentration decreased significantly in both groups, but with smaller decreases in rifampicin participants (global p=0·001, appendix p 12). Two (1%) participants who received rifampicin developed rifampicin-resistant *S aureus* bacteraemia 7 days and 42 days after randomisation (p=0·24; appendix p 7); one participant had rifampicin-resistant *S aureus* isolated from another sterile site after randomisation (a pacemaker lead removed 4 h after the first trial drug dose, see appendix p 7).

By week 12, 101 (27%) participants in the rifampicin group versus 94 (24%) in the placebo group had 112 versus 116 SAEs (HR 1·21, 95% CI 0·92–1·61, log-rank p=0·17; appendix pp 20, 21). Two participants with pre-existing liver disease who received rifampicin had non-fatal hepatic failure (appendix p 7). No between-group differences in changes in alanine transaminase (global p=0·18; appendix p 12) or alkaline phosphatase (global p=0·11) were observed (appendix p 13). Bilirubin significantly increased in the rifampicin group at day 3 (p<0·0001; global p<0·0001; appendix p 12).

129 (35%) participants in the rifampicin group versus 131 (34%) in the placebo group had 209 versus 193 grade 3–4 AEs (HR 1·12, 95% CI 0·88–1·43, log-rank p=0·36; table 4). Most notable was a trend towards more renal grade 3–4 AEs with rifampicin, which occurred in 19 (5%) versus nine (2%; p=0·053) participants, with 17 versus six being acute kidney injury. 63 (17%) participants who received rifampicin versus 39 (10%) who received placebo had 89 versus 52 antibiotic-modifying AEs (subdistribution HR 1·78, 95% CI 1·20–2·65, log-rank p=0·004, appendix pp 20, 26). Gastrointestinal disorders (24 *vs* eight; p=0·003) and renal or urinary disorders (eight *vs* one; p=0·02) were more common with rifampicin than placebo. 24 (6%) participants in the rifampicin group versus six (2%) in the placebo group had drug interactions (p=0·0005); 13 versus four led to discontinuation of trial drug (p=0·03), 14 versus three led to grade 1–2 AEs (p=0·006), and five versus two to grade 3–4 AEs (p=0·27).

**Table 4:** Summary of grade 3–4 adverse events

	**Placebo (n=388)**		**Rifampicin (n=370)**		**Total (N=758)**		p value*
	Patients with event (%)	Total events	Patients with event (%)	Total events	Patients with event (%)	Total events	
Any	131 (34%)	193	129 (35%)	209	260 (34%)	402	0·76
Infections and infestations	45 (12%)	53	40 (11%)	48	85 (11%)	101	0·82
Cardiac disorders	15 (4%)	17	6 (2%)	8	21 (3%)	25	0·08
Vascular disorders	7 (2%)	7	5 (1%)	5	12 (2%)	12	0·77
Respiratory, thoracic, and mediastinal disorders	16 (4%)	17	10 (3%)	11	26 (3%)	28	0·32
Gastrointestinal disorders	21 (5%)	24	29 (8%)	40	50 (7%)	64	0·19
Hepatobiliary disorders	0 (0%)	0	3 (1%)	3	3 (<1%)	3	0·12
Skin and subcutaneous tissue disorders	7 (2%)	7	5 (1%)	5	12 (2%)	12	0·77
Musculoskeletal and connective tissue disorders	2 (1%)	2	0 (0%)	0	2 (<1%)	2	0·50
Renal and urinary disorders	9 (2%)	9	19 (5%)	20	28 (4%)	29	0·053
Neoplasms benign, malignant, and unspecified (including cysts and polyps)	7 (2%)	7	11 (3%)	12	18 (2%)	19	0·34
Reproductive system and breast disorders	0 (0%)	0	1 (<1%)	1	1 (<1%)	1	0·49
Congenital, familial, and genetic disorders	1 (<1%)	1	0 (0%)	0	1 (<1%)	1	1·00
General disorders and administration site conditions	11 (3%)	11	12 (3%)	12	23 (3%)	23	0·83
Investigations	6 (2%)	6	11 (3%)	16	17 (2%)	22	0·22
Injury, poisoning, and procedural complications	6 (2%)	6	5 (1%)	5	11 (1%)	11	1·00
Surgical and medical procedures	0 (0%)	0	1 (<1%)	1	1 (<1%)	1	0·49
Blood and lymphatic system disorders	3 (1%)	3	5 (1%)	6	8 (1%)	9	0·50
Metabolism and nutrition disorders	3 (1%)	3	5 (1%)	6	8 (1%)	9	0·50
Psychiatric disorders	5 (1%)	5	5 (1%)	6	10 (1%)	11	1·00
Nervous system disorders	11 (3%)	14	4 (1%)	4	15 (2%)	18	0·12
Eye disorders	1 (<1%)	1	0 (0%)	0	1 (<1%)	1	1·00

* Fisher's exact test.

At least one individual was unmasked for 14 participants (nine rifampicin, five placebo). In two cases, a non-trial physician or ward pharmacist were unmasked for participant safety, and in three cases, the research nurse was unmasked.

At the final 12-week visit, physicians and participants were asked which treatment they believed had been administered. 203 (84%) of 243 physicians of participants randomly assigned to the rifampicin group reported that they genuinely had no idea versus 249 (89%) of 279 physicians of participants in the placebo group (p=0·08); 32 (13%) and 17 (6%) identified the correct allocation. By contrast, 113 (57%) of 199 participants randomly assigned to the rifampicin group reported that they genuinely had no idea versus 159 (69%) of 229 participants randomly assigned to placebo (p=0·007); 72 (36%) and 35 (15%) identified the correct allocation.

## Discussion

This large, multicentre, randomised, double-blind, placebo-controlled trial, including 758 adults with *S aureus* bacteraemia, aimed to establish whether rifampicin added to standard backbone antibiotics for up to 14 days reduced bacteriologically confirmed treatment failure or disease recurrence, or death, by 12 weeks. We found that although rifampicin did not have a significant effect on any of the composite primary or secondary efficacy measures, including mortality, duration of bacteraemia, or development of rifampicin-resistant *S aureus*, it was associated with a small but significant reduction in bacteriologically and clinically defined disease recurrences.

Our trial highlights the severity and heterogeneity of *S aureus* bacteraemia. Participants were mostly older adults with comorbidities, 9% of whom were enrolled in an intensive care unit. Most (64%) infections were acquired in the community, and 6% were caused by MRSA, reflecting substantial improvements in prevention and control of hospital-acquired *S aureus* and MRSA infection in the UK over the past decade, and the consequent decline in infections.^14^ A deep infection focus was present at baseline in 301 (40%) participants—around half with endocarditis, orthopaedic or intravascular devices, or osteoarticular infections—and 139 (18%) had no established infection focus. Therefore, a substantial proportion had what could be considered as uncomplicated disease.

The choice and duration of backbone antibiotics varied substantially between participants, but 82% received flucloxacillin and 50% received a glycopeptide at some point during their primary treatment. The choice of first-line anti-staphylococcal penicillin for the treatment of MSSA infections varies by country. In the UK and Australia, flucloxacillin is used; whereas, in the USA, other agents, such as nafcillin and cloxacillin, are preferred. There is no evidence to support clinically relevant differential anti-staphylococcal activity between these antibiotics,^15^, ^16^ and we therefore believe our results are generalisable across countries, regardless of their chosen anti-staphylococcal penicillin. The use of other antibiotics (including open-label rifampicin) and the total duration of active antibiotic therapy (median 29 days) was similar between randomised groups. Fewer rifampicin-treated than placebo-treated participants were restarted on antibiotics after the primary treatment course, possibly reflecting the lower recurrence rate in this group.

Planned subgroup analysis suggested the effect of rifampicin might have varied according to antibiotics used at randomisation, with any benefit restricted to those with meticillin-sensitive infection treated with flucloxacillin alone. The clinical significance of this result is uncertain. The effect was lost if flucloxacillin was used with vancomycin or another antibiotic, or if the subgroups were defined by antibiotic class. With 20 subgroups analysed, one statistically significant association might have occurred by chance.

Our results refute the hypothesis that adjunctive rifampicin enhances *S aureus* killing in blood and thereby reduces the risk of dissemination and death.^17^ Bacterial clearance in blood was similar in the two treatment groups, and all-cause mortality was unaffected by rifampicin. Even deaths adjudicated as definitely or probably due to *S aureus* (50%) were not reduced by rifampicin. However, 12-week all-cause mortality (15%) was substantially lower than was reported in a large multicentre case-series (29%),^2^ although was similar to the trial of daptomycin in *S aureus* bacteraemia.^18^ This similarity might be explained by some severely unwell patients being unable to enter the trial, including the 129 patients who were not enrolled because they either died or were considered too unwell for active treatment (figure 1). Participants might also have benefited from regular infection specialist consults, mandatory for the trial and associated with improved *S aureus* bacteraemia outcomes.^19^ It highlights the importance of basing sample size calculations on previous trials wherever possible.

Our findings provide some support for another hypothesis—that rifampicin enhances the sterilisation of deep infection foci and thus reduces disease recurrences.^20^ The small but statistically significant reduction in recurrences in the rifampicin group indicates the drug had some biological activity, although its clinical significance is debatable. The numbers needed to treat to prevent bacteriologically and clinically defined disease recurrences were 29 and 26, and both short-term and long-term mortality was unaltered (Figure 2, Figure 3). This modest effect needs to be balanced against the complications of rifampicin use and its toxicity. 306 (11%) of 2896 screened patients were not enrolled because of predicted drug interactions or pre-existing liver disease, and although the number of participants who had SAEs was similar between the groups, there were significantly more antibiotic-modifying AEs and drug interactions in the rifampicin group. AEs were predominantly gastrointestinal disorders and renal impairment. In addition, the independent, masked review committee adjudicated that recurrences were more commonly caused by failure to recognise or remove the primary infection focus than a failure of antibiotic treatment (table 3). Therefore, rifampicin might assist in the sterilisation of deep *S aureus* infection foci and prevent a few recurrences, but it does not replace the need to define and, when possible, drain or remove the infection focus.

The strengths of the trial are its placebo-controlled, multicentre and pragmatic design, which provides clinically relevant, generalisable findings. It is also the largest trial to date that examines *S aureus* bacteraemia treatment. Its limitations reflect the many challenges of performing trials in acutely unwell patients with severe bacterial infections.^21^ Disease severity and heterogeneity, and the requirement to randomly assign patients within 96 h of the start of antibiotic therapy, increased the proportion of ineligible patients and slowed recruitment. Only 770 (27%) of those screened were enrolled and 664 (31%) not enrolled had received more than 96 h of antibiotics. 232 (11%) subjects were not enrolled because rifampicin was considered mandatory; although this information was only collected as an exclusion criteria without additional details, anecdotal evidence indicated many of these patients had prosthetic-related infections. These exclusions might have reduced the clinical effect of rifampicin and the relevance of the findings to those with bacteraemia associated with infected prostheses who might benefit more from rifampicin.^9^ Additional limitations include possible heterogeneity arising from the range of recruited participants per centre (1–164), and the observation that approximately 30% of patients either initiated open-label rifampicin or stopped the blinded trial drug early, predominantly for drug–drug interactions or AEs. However, these changes are also likely to happen in normal clinical practice. Additionally, vital status or signs and symptoms, or both, could not be ascertained in approximately 9% of patients at the 12-week follow-up.

Although few participants had MRSA bacteraemia, we found no evidence of heterogeneity in the effect of rifampicin in this subgroup, which, if anything, did worse with rifampicin than placebo (figure 2). Similarly, although treatment with rifampicin was started anywhere between 0 h and 96 h after active antibiotics (meaning some patients could theoretically have had a clinically meaningful delay in starting rifampicin, which could have affected efficacy), we found no evidence of such an effect in subgroup analyses, based on time from randomisation to initiation of rifampicin or placebo as a categorical or continuous factor (figure 2), supporting wider generalisability. Non-significant trends toward differences in other subgroups of particular interest, including infections with a deep focus, which comprised approximately 40% of the total patient population, need to be interpreted carefully given the large numbers of significance tests done.^22^

On the basis of a small systematic review,^11^ the trial was originally powered to detect an absolute difference of 10% in bacteriological treatment failure or death (from 35% to 25%) and a 7% absolute reduction in mortality from 16% to 9%. Although slow recruitment meant the sample size was reduced, the 770 participants included are more than double the number in the biggest previous trial in *S aureus* bacteraemia,^23^ and is a 50% increase in the total numbers of cases recruited in randomised trials over the past 50 years. The 95% CIs around our estimates of the difference between rifampicin and placebo lie within 7·5%, which is smaller than the 10% non-inferiority margins recommended by licensing authorities for antibiotic trials and commonly used in other infections, such as HIV. The use of an active comparator would have conclusively shown non-inferiority of rifampicin. Although designed to test superiority of rifampicin, the trial thus provides convincing evidence of non-inferiority of rifampicin to placebo; that is, convincing evidence of absence of benefit. A small proportion (13%) of participants used open-label rifampicin in the placebo group, but per-protocol analyses confirmed this well estimated absence of benefit of rifampicin over placebo.

In summary, adjunctive rifampicin did not improve outcomes from *S aureus* bacteraemia, with the exception of a modest reduction in disease recurrence. Given rifampicin had no effect on short-term or long-term mortality, substantially complicated other drug treatment, and widespread use risks increasing resistance among *S aureus* and other bacteria (eg, *Mycobacterium tuberculosis*), we consider that adjunctive rifampicin provides no overall benefit over standard antibiotic therapy in adults with *S aureus* bacteraemia.
