# Supplementary material for: Adjunctive rifampicin for Staphylococcus aureus bacteraemia (ARREST): a multicentre, randomised, double-blind, placebo-controlled trial
Source: Lancet. 2018 Feb 17;391(10121):668–78. doi: 10.1016/S0140-6736(17)32456-X (PMC5820409; doi:10.1016/S0140-6736(17)32456-X)
Supplement: Supplementary appendix [file mmc1.pdf]

THE LANCET

Supplementary appendix

This appendix formed part of the original submission and has been peer reviewed. We post it as supplied by the authors.

Supplement to: Thwaites GF, Scarborough M, Szubert A, et al. Adjunctive rifampicin for Staphylococcus aureus bacteraemia (ARREST): a multicentre, randomised, double-blind, placebo-controlled trial. *Lancet* 2017; published online Dec 14. http://dx.doi.org/10.1016/S0140-6736(17)32456-X.

Supplementary material

List of investigators....2

Supplementary Methods 4

(a) Further details of endpoint ascertainment 4

(b) Failure and recurrence endpoint definition 4

(c) Statistical methods....4

(d) Subgroup analyses....5

(e) Blinded endpoint review committee....6

Supplementary Results....7

(a) Per-protocol analysis of the primary endpoint 7

(b) Further details of rifampicin-resistant *S. aureus* 7

(c) Hepatic SAEs 7

Figure S1 Reported missing doses before permanent discontinuation....8

Figure S2a Five priority subgroup analyses for bacteriological failure/recurrence or death through 12 weeks (primary endpoint) 9

Figure S2b Twelve other subgroup analyses for bacteriological failure/recurrence or death through 12 weeks (primary endpoint) 10

Figure S3 Persistence of bacteraemia....11

Figure S4 Changes in laboratory test results over 2 weeks from randomisation (a) CRP, (b) ALT, (c) alkaline phosphatase and (d) bilirubin....12

Table S1 Additional characteristics at randomiation 14

Table S2 Initial infection focus in participants who received open label rifampicin at any point during 12 weeks follow-up……16

Table S3 Infection focus management....16

Table S4 Primary and secondary causes of deaths through 12 weeks....17

Table S5 Summary of SAEs, Grade 3/4 and antibiotic-modifying adverse events....20

Table S6 Serious adverse events....21

Table S7 Drug-modifying adverse events....26

Supplementary References....29

List of investigators

The ARREST trial group consists of:
Participating Centres:
Oxford University Hospitals NHS Trust: M Scarborough, M Kamfose, A de Veciana, NC Gordon, L Peto, G Pill, T Clarke, L Watson, B Young, D Griffiths, A Vaughn, L Anson, E Liu, S Perera, L Rylance-Knight, C Cantell, R Moroney.
Guy's and St Thomas' NHS Foundation Trust: JD Edgeworth, G Thwaites, K Bisnauthsing, A Querol-Rubiera, C Gibbs, A Patel, C Hemsley, AL Goodman, D Wyncoll, J Biswas, J Fitzpatrick, L Roberts, J Millard, N Stone, A Cape, L Hurley, C Kai Tam.
The Royal Liverpool and Broadgreen University Hospitals NHS Trust: E Nsutebu, M Hoyle, K Maitland, L Trainor, H Reynolds, J Harrison, J Anson, J Lewis, J Folb, L Goodwin, N Beeching, S Dyas, H Winslow, E Foote, P Roberts, P Natarajan, A Chrdle. M Fenech, H Allsop.
Plymouth Hospitals NHS Trust: R Tilley, R Austin-Hutchison, L Barrett, K Brookes, L Carwithen, A Conbeer, R Cunningham, C Eglinton, R Fok, H Gott, S Hughes, L Jones, M Kalita, A King, L March, M Marner, T Mynes, A Plant, S Price, J Sercombe, A Stolton, M Wallis, M West, J Westcott, C Williams, R Wosley, L Yabsley.
Sheffield Teaching Hospitals NHS Foundation Trust: J Greig, L Butland, J Sorrell, T Mitchell, A Alli, J Meiring, B Masake, C Rowson, L Smart, L Makey, S Moll, J Cunningham, K Ryalls, K Burchall, J Middle, Y Jackson, D Swift, J Cole, B Subramanian, F Okhuoya, M Edwards, C Bailey, R Warren, G Islam, M Ankcorn, S Birchall, P Jones, J Humphries, S Booth, C Evans.
Portsmouth Hospitals NHS Trust: S Wyllie, A Flatt, L Strakova, M Hayes, S Valentine, C James, M Wands, N Cortes, N Khan, R Porter, Z Martin, K Yip, H Preedy, H Chesterfield, T Dobson, C Walker.
Brighton and Sussex University Hospitals NHS Trust: M Llewelyn, A Dunne, L Latter, A Porges, J Price, J Paul, L Behar, L Robinson, A Murray, J Fitzpatrick, T Sargent, C Ridley, L Ortiz-Ruiz de Gordoa, D Gilliam, C McPherson, S Matthews, E Foreman, R Jarghese, A Beddoe, S Martin, S Shaw, D Wlazly, M Cole, A Gihawi, K Cole.
Cambridge University Hospitals NHS Foundation Trust: ME Török, T Gouliouris, L Bedford, RB Saunderson, I Mariolis, R Bousfield, I Ramsay, D Greaves, S Aliyu, K Cox, L Mlemba, L Whitehead, N Vyse, M Bolton
South Tees Hospitals NHS Foundation Trust: J Williams, P Lambert, D Chadwick, K Baillie, M Cain, R Bellamy, J Wong, J Thompson, H Vassallo, A Skotnicka, A Boyce, A Donnelly.
University College London Hospitals NHS Foundation Trust: P Wilson, G FitzGerald, V Dean, K Warnes, A Reyes, S Rahman, L Tsang, J Williams, S Morris-Jones.
Royal Free London NHS Foundation Trust: S Hopkins, E Witness, O Brady, E Woodford, T Pettifer, A McCadden, B Marks, S Collier, D Mack, S Warren, C Brown, A Lyons, S Taiyari, S Mepham, A Sweeney, L Brown.
Royal Devon and Exeter NHS Foundation Trust: C Auckland, A Potter, J Mandiza, M Hough, S Williams, C Renton, F Walters, M Nadolski, M Hough, A Evans, P Tarrant, S Williams, K Curley, S Whiteley, J Halpin, M Hutchings, S Todd, C Lohan, T Chapter, E Folland, A Colville, K Marden, M Morgan, R Fok, R Porter, M Baxter.
The Leeds Teaching Hospital NHS Trust: J Minton, S Rippon, M Cevik, J Chapman, T Kemp, R Vincent, D Osborne, T Platt, J Calderwood, B Cook, C Bedford, L Galloway-Browne, N Abberley, K Attack, J Allen.
Aintree University Hospital NHS Foundation Trust: P Lal, M Harrison, S Stevenson, C Brooks, P Harlow, J Ewing, S Cooper, R Balancio-Tolentino, L O'Neil, R Tagney, D Shackcloth.
St George's Healthcare NHS Trust: T Planche, J Fellows, R Millett, J Studham, C de Souza, G Howell, H Greaves, E Foncel, R Kurup, J Briggs, M Smith, C Suarez, G Sorrentino, A Scobie, A Houston, F Ahmad, A Breathnach, R Chahuan, K Wilkins.
Blackpool Teaching Hospitals NHS Foundation Trust: A Guleri, N Waddington, R Sharma, P Flegg, V Kollipara, M Alam, A Potter, S Donaldson, C Armer, J Frudd.
King's College Hospital NHS Foundation Trust: D Jeyaratnam, M Joy, A Mathews, SK Glass, A Ajayi, A Fife, S Qaiser, S Sheehan, S Muñoz Villaverde, NO Yogo, I De Abreu, G Notcheva, J Flanagan, C Watson, E Sais, A Adedayo, V Chu, G Shaw, MA Graver, R Palmer, D Palmer, S Haile, J Gordon, C Kai Tam, Mandar K, W Szypura.
Heart of England NHS Foundation Trust: N Jenkins, J Marange, V Shabangu, K Moore, J Lyons, M Munang, M Sangombe, E Moran, A Hussain.
University Hospitals of Leicester NHS Trust: M Wiselka, A Lewszuk, S Batham, K Ellis, L Bahadur, H White, M Pareek, A Sahota, S Coleman, H Pateman, A Kotecha, C Sim, A Rosser.
County Durham and Darlington NHS Foundation Trust: D Nayar, J Deane, R Nendick, C Aldridge, A Clarke, M Wood, A Marshall, L Stephenson, T Matheson-Smith, J Sloss, K Potts, J Malkin, L Ftika, V Raviprakash.
University Hospital Southampton NHS Foundation Trust: J Sutton, A Malachira, M Kean, K Criste, K Gladas, C Andrews, C Hutchison, E Adams, J Andrews, B Romans, N Ridley, M Ekani, J Mitchell, N Smith, T Clark, S Glover, R Reed, T Yam, H Burton, R Said.

Wirral University Teaching Hospital NHS Foundation Trust: D Harvey, A Janvier, R Jacob, C Smalley, A Fair.
Dartford & Gravesham NHS Trust: A Gonzalez-Ruiz, S Lord, K Ripalda, H Wooldridge, L Cotter, G Cardoso, E Strachan, G Kaler, A Mohamoodally, E Lawrence, Z Prime, R Abrahams.
The Newcastle upon Tyne Hospitals NHS Foundation Trust: DA Price, L Rigden, L Shewan, K Cullen, I Emmerson, K Martin, H Wilson, C Higham, K L Taylor, E Ong, B Patel, H Bond, J Gradwell, J Widdrington.
North Cumbria University Hospitals: C Graham, S Thornthwaite, S Prentice, U Poultney, H Crowther, H Fairlamb, E Hetherington, C Brewer, S Banerjee, C Hamson, A McSkeane.
Bradford Teaching Hospitals NHS Foundation Trust: P McWhinney, P Sharratt, J Thorpe, S Kimachia, H Wilson B Jeffs, L Masters, J Wilson, J Platt, L Burgess.
Salford Royal NHS Foundation Trust: P Chadwick, A Jeans, C Keatley, A Moran, Z Swann, K Pagett, A Peel, J Howard.
Royal United Hospital Bath NHS Trust: S Meisner, K Maloney, A Masdin, L Wright.
Hull and East Yorkshire Hospitals NHS Trust: G Barlow, S Crossman, V Lowthorpe, E Moore, P Moss, A Parkin, A Wolstencroft, B Warner, C Tarbotton, A Eyre, A Anderson, T Burdett, A Driffill.

Trial Coordination and Oversight - MRC CTU at UCL, London: AS Walker, F Hudson, A Szubert, J Cairns, D Ward, H Webb, C Russell, B Jackson, D Otiko. **Data Management Systems**: C Borg, L Masters, Z Islam, C Diaz, D Johnson

Trial Steering Committee: (Independent) A Martineau (Chair), A Kaasch, G Scott, J Bostock. (Team) G Thwaites, AS Walker, G Barlow, S Hopkins.

Data Monitoring Committee: D Lalloo (Chair), M Wilcox, D Altman.

Independent Endpoint Review Committee: T Peto, G Cooke.

Supplementary Methods

(a) Further details of endpoint ascertainment

SAEs were defined following the International Committee for Harmonization as events which led to death, were life-threatening, caused or prolonged hospitalisation (excluding elective procedures), caused permanent disability, or were other medical conditions or with a real, not hypothetical risk of one of the previous categories.

(b) Failure and recurrence endpoint definition

Bacteriologically confirmed treatment failure was defined in the protocol as symptoms and signs of infection for longer than 14 days from randomisation with the isolation of same strain of *S. aureus* (confirmed by genotyping) from a sterile site (e.g. blood, joint fluid, pus from tissue). Disease recurrence was defined in the protocol as the isolation of the same strain of *S. aureus* from a sterile site after at least 7 days of apparent clinical improvement.

A substantial proportion of bacteriological failure/recurrences did not have both baseline and failure/recurrence isolates stored (17 (61%) of 28 failures/recurrences where *S. aureus* was isolated from a sterile site). In order to avoid excluding a substantial proportion of potential primary endpoints, the statistical analysis plan specified that the primary analysis would include all bacteriologically-confirmed failures and recurrences (i.e. without restricting to the same strain).

Same strain was defined by whole-genome-sequencing using Illumina technology on the basis of 40 single nucleotide variants between baseline and failure/recurrence isolates.

Clinically-defined failure and recurrence followed the same definitions, but without the requirement for microbiological confirmation.

(c) Statistical methods

Analyses followed the principle of intention-to-treat including all follow-up regardless of changes to treatment. The Statistical Analysis Plan pre-specified that any patient who was randomised in error (defined as realising that the patient should not have been randomised before taking blinded study drug and not ever taking study drug) and hence not followed up would be excluded. The blinding means that there was no possibility that knowledge of randomised allocation affected this judgement about what was an error. Any participants who were randomised in good faith (i.e. not by mistake) but never took study drug were included in all analyses.

Time-to-event analyses measured time from randomisation. Analyses of clinical outcomes censored at the earliest of 12 weeks from randomisation and the last clinical information. Analyses of mortality censored at the earliest of the timescale being considered (2 weeks, 12 weeks) or last vital status information (including that ascertained at trial closure through the National Health Service records).

The primary analyses were unstratified because the randomisation stratification factor (centre) was expected to have some small strata and participants in these strata might then not contribute to comparisons. Results from secondary stratified analyses (stratified logrank test and stratified Cox regression) were very similar (data not shown). Lost-to-follow-up was defined as not having been assessed in person or by telephone within a [-1,+8] week window of the 12 week final visit by a trial clinician and not having information on whether or not signs/symptoms of *S. aureus* were present (e.g. from the patient's General Practitioner).

Primary analysis of the primary endpoint included all randomised participants other than those considered randomised in error (following the statistical analysis plan): secondary analysis of the primary endpoint was to exclude those (expected <1%) who were subsequently identified as having had a rifampicin resistant *S. aureus* bacteraemia on susceptibility testing. As no patients were identified after randomisation as having had a rifampicin resistant *S. aureus* bacteraemia at enrolment, this analysis was identical to the primary analysis. In the statistical analysis plan (but not the protocol), a per-protocol analysis was also specified for the primary endpoint, including all participants in the primary intention-to-treat analysis who received active/placebo for ≥80% of days from start of trial drug to earliest of: 14 days subsequently/death/discontinuation of active antibiotics (not including trial drug).

Safety analyses included all data between randomisation and 12 weeks post-randomisation (inclusive). Non-fatal events related to *S. aureus* bacteraemia were not considered AEs/SAEs in the protocol.

Analyses of all time-to-event outcomes which did not include all-cause mortality used competing risks methods. These estimated the probability of the event (analogous to Kaplan-Meier) using cumulative incidence functions, and estimated the effect of randomised group on the subdistribution hazard corresponding to the cumulative incidence function.[1] These analyses were only conducted unstratified, as stratification is not possible with the estimating equation approach used for estimation.

C-reactive protein (CRP) and liver function test results were compared between randomised groups over time using generalised estimating equations (GEE) (normal distribution, independent correlation structure) with randomised group, adjusting for the stratification factor, baseline values and scheduled visit week as categorical independent variables and interaction between baseline values and scheduled visit week. The closest measurement to each scheduled visit date within equally spaced windows was used as the measurement at each scheduled visit. The midpoint between two scheduled assessment days was taken as belonging to the latter window. Where there were two values within one of these equally spaced windows, but both equidistant from the nominal assessment day, the later value was used. Analyses were based on observed data. To account for CRP values above limit of quantification in one centre (that is, CRP only reported as >156 mg/L if above this threshold), mean CRP (Table 1, Figure S3(a)) was estimated using normal interval regression. For analyses of change from baseline, these values were assumed equal to the limit of quantification.

For blood cultures, baseline (used to define baseline resistance/susceptibility) was defined as the closest up to and including day 0, and up to one day post-randomisation providing this was on or before date of start of trial drug. Cultures prior to randomisation were used in preference to cultures the same number of days after randomisation, but on or before the date of start of trial drug. As eligibility was based on the screening positive blood culture, and because the intention was to characterise persisting bacteraemia, baseline bacteraemia included cultures on day-one where a culture on the day of or on the day prior to randomisation was not available. For duration of bacteraemia, baseline was defined as the closest up to and including day 0 within the preceding day, and up to one day post-randomisation.

For laboratory measurements (eg CRP), baseline was defined as the closest up to and including day 0 within the preceding 4 days, and up to one day post-randomisation providing this was on or before date of start of trial drug. Measurements prior to randomisation were used in preference to measurements the same number of days after randomisation, but on or before the date of start of trial drug.

The protocol and statistical analysis plan specified that the primary outcome (bacteriologically-confirmed failure/recurrence or death) would be analysed using time-to-event methods as above. The sample size calculation treated this outcome as binary, in order to produce a conservative estimate of sample size given uncertainties in the underlying assumptions, and since all patients were to be followed for a fixed 12 week period (that is, no additional power was gained from longer follow-up in some patients).

(d) Subgroup analyses
Subgroup analyses were conducted to assess consistency of effects across different participant characteristics. The primary method of assessing subgroup effects was an interaction test within a Cox proportional hazards regression. For the continuous factors we used both categorisation and natural cubic splines (five knots at the 10th, 25th, 50th, 75th, and 90th centiles; four knots at the 10th, 33rd, 67th, and 90th centiles for Charlson comorbidity index score (as 10th and 25th centiles identical)) to test for interactions. Subgroup analyses were conducted unstratified to avoid losing information from small strata with no events in one randomised group. No formal adjustment for multiple testing was made for subgroup analyses.

We pre-specified in the protocol twelve subgroup analyses for the primary endpoint; namely time from initiation of antibiotics to initiation of randomised treatment, time from randomisation to initiation of randomised treatment, initial oral randomised treatment frequency (once vs twice daily), initial treatment with oral trial drug only or regimen containing IV trial drug, class of primary antibiotic treatment, other antibiotic adjuncts (e.g. gentamicin), MRSA/MSSA, IV catheter-associated infection/other, deep focus/no deep focus, endocarditis/no endocarditis, age and CRP (terciles).

The statistical analysis plan included 6 additional subgroup analyses, but prioritised the subgroup analyses as follows (*=in protocol).

1. *Time from initiation of first active antibiotic treatment to initiation of randomised treatment (0-24, >24-48, >48-72, >72 hours)
1. *Class of initial antibiotic treatment, and according to individual drugs where these are used by >10% of the trial population

1. *MRSA/MSSA
1. *IV catheter (central/peripheral venous line)/implanted vascular device-associated infection vs.other (based on portal of entry)
1. *Deep focus (implanted vascular device, native/prosthetic heart valve, native/prosthetic joint, deep tissue infection/abscess)/no deep focus (based on foci of infection)
1. *Endocarditis (main focus/foci of infection at time first positive blood culture taken = native heart valve/prosthetic heart valve)/no endocarditis
1. *Foci of infection known/not known
1. *Age (terciles)
2. *Initial oral randomised treatment frequency (once vs twice daily)
2. *Initial treatment with oral trial drug only or regimen containing IV trial drug
2. *Whether gentamicin was administered between first positive blood culture and 48 hours post-randomisation, regardless of activity
2. Whether any active antibiotic other than that first administered (excluding trial drug), trial drug and gentamicin was administered between first positive blood culture and 48 hours post-randomisation (yes vs no)
2. *Baseline CRP (terciles)
2. Charlson comorbidity index score (0, 1-2, 3-4, ≥5)
3. Time from randomisation to initiation of randomised treatment (0-4, >4-12, >12-24, >24 hours)
3. Community, healthcare associated and nosocomial acquisition
3. Calendar year of randomisation
3. Baseline neutrophils (terciles)

We also considered additional exploratory subgroups defined by initial total daily dose (600 vs 900 mg), and whether or not the patient was bacteraemic at randomisation, leading to 20 subgroups in total.

(e) Blinded endpoint review committee

The blinded independent review committee consisted of two infectious disease physicians with experience in acute/general medicine (Professor Tim Peto, Oxford; Dr Graham Cooke, Imperial; see acknowledgements). Potential failures/recurrences were identified through questions regarding signs and symptoms of ongoing or new S. aureus infection on routine case record forms, and by electronic searching of new or ongoing foci of infection being reported, and S. aureus isolated from any microbiological specimen. For all such potential failures/recurrences a structured clinical narrative was completed by the site physician and approved by the site PI. All reported failures, recurrences and deaths were then adjudicated using standardised proformas by the committee without knowledge of randomised allocation.

Supplementary Results

(a) Per-protocol analysis of the primary endpoint
242 (65.4%) rifampicin versus 290 (74.7%) placebo were included in the per-protocol population (received active rifampicin/placebo for ≥80% of days from start of trial drug to earliest of: 14 days subsequently/death/discontinuation of active antibiotics (not including trial drug)).

By 12 weeks, 39 (16.1%) rifampicin versus 49 (16.9%) placebo experienced bacteriological failure/recurrence or died (absolute risk difference (RD)=-0.8% (95% CI –7.3,+5.6); hazard ratio (HR)=1.00 (0.65,1.52) p=0.99).

An exploratory post-hoc analysis was also done additionally excluding participants in either group who started open-label rifampicin at any time during follow-up. 225 (60.1%) rifampicin versus 262 (67.5%) placebo were included in this post-hoc per-protocol population. By 12 weeks, 37 (16.4%) rifampicin versus 37 (14.1%) placebo experienced bacteriological failure/recurrence or died (absolute risk difference (RD)=2.3% (95% CI –4.3,+8.8); hazard ratio (HR)=1.23 (0.78,1.93) p=0.38).

(b) Further details of rifampicin-resistant *S. aureus*
Two (0.5%) rifampicin participants developed new rifampicin-resistant *S. aureus* bacteraemia (p=0.24). One occurred on day 7 (followed by rifampicin discontinuation on day 11 and bacteriological failure on day 14); the other on day 42 (prescribed 14 days rifampicin; bacteriological recurrence on day 42).

One additional participant had rifampicin-resistant *S. aureus* isolated from a permanent pacemaker wire removed on day 1 (within 4 hours of the first dose of trial drug). The screening blood culture had isolated a rifampicin-sensitive *S. aureus*. Further blood cultures were sterile for the remainder of follow-up. Following whole genome sequencing, the rifampicin resistant pacemaker isolate was 11 single nucleotide polymorphisms from the screening isolate and another isolate taken from the pacemaker on day-1, whereas these latter two isolates did not differ genetically, suggesting a diversity between isolates of more than 3 days in origin, and thus suggesting that the patient had a mixed infection with both rifampicin-resistant and rifampicin-susceptible strains that was not detected at screening.

Eighty-eight patients in the rifampicin group had positive blood cultures at enrolment (group comparison given in **Figure S3**). Of these 88, only one failed bacteriologically, none had bacteriological recurrence and none developed rifampicin-resistant infection. Eight failed clinically (including the one who failed bacteriologically) and two had clinical recurrence.

(c) Hepatic SAEs
Two rifampicin participants experienced hepatic SAEs.

One 47-year old female required prolongation of hospitalisation for acute hepatic failure (grade 3) with raised INR (grade 2), ascites (grade 3) and acute renal failure (grade 3) which developed on ICU following 5 days rifampicin (900mg daily) with flucloxacillin. The participant had pre-existing Hepatitis C and chronic liver disease. Acute hepatic and renal failure was considered to have been triggered by sepsis. The participant recovered.

One 51-year-old female required prolongation of hospitalisation for decompensated liver disease (grade 3) with ascites (grade 3) following 14 days rifampicin (initially on 900 mg daily) with flucloxacillin). The participant did not mention liver disease at screening/enrolment and there was nothing in her medical notes regarding any past history of liver problems. When she developed decompensated liver disease with ascites, it was discovered that she had had a previous diagnosis of non-alcoholic steatosis (NASH) at another hospital several years previous, but was no longer under follow up. The participant recovered.

Figure S1 Reported missing doses before permanent discontinuation

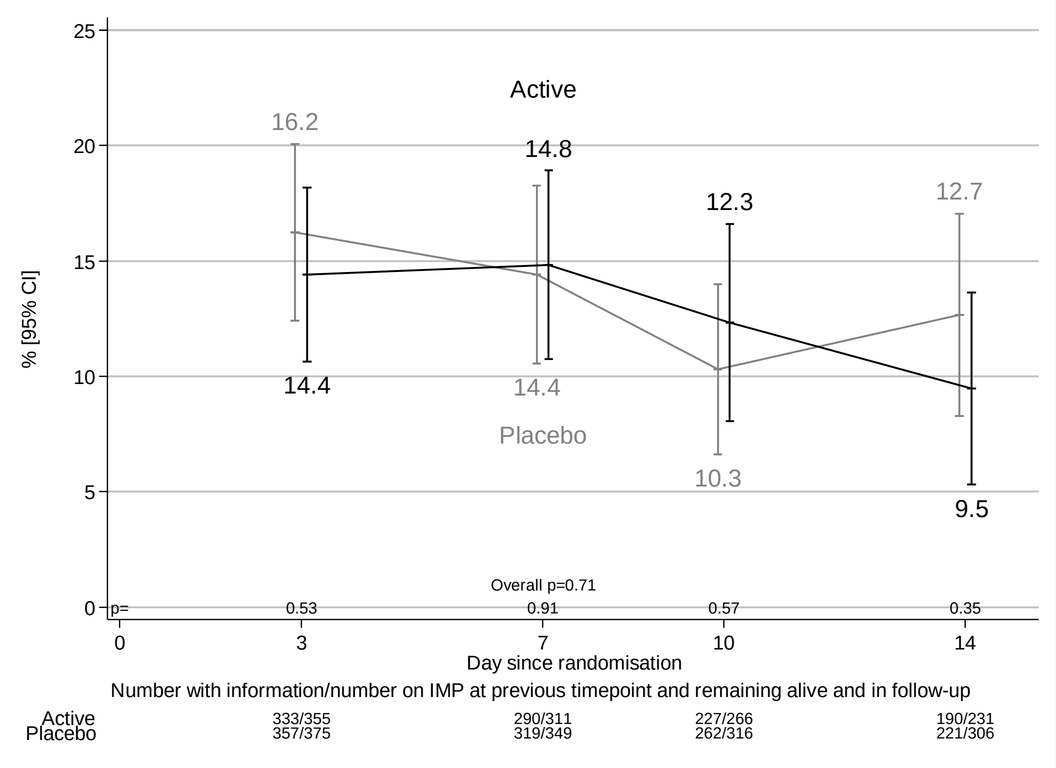

Figure S2a Five priority subgroup analyses for bacteriological failure/recurrence or death through 12 weeks (primary endpoint)

	Placebo	Active	Active:placebo HR [95% CI]	Interaction p
INITIAL ACTIVE ANTIBIOTIC (CLASS-LEVEL)				
(1) MSSA: penicillin(s) / cephalosporin(s) / carbapenem(s)	54/266(20.3%)	38/245(15.5%)	0.79 [0.52, 1.20]	0.35
(2) MSSA: glycopeptide +/- (1)	6/52(11.5%)	8/58(13.8%)	1.18 [0.41, 3.41]	
(3) MSSA: (1) and other	5/33(15.2%)	6/32(18.8%)	1.40 [0.43, 4.58]	
(4) MSSA: other	3/16(18.8%)	1/9(11.1%)	0.69 [0.07, 6.67]	
(5) MRSA	3/21(14.3%)	9/26(34.6%)	2.74 [0.74, 10.15]	
INITIAL ACTIVE ANTIBIOTIC (ANTIBIOTIC-LEVEL)				
MSSA: flucloxacillin only	38/191(19.9%)	16/176(9.1%)	0.45 [0.25, 0.81]	0.01
MSSA: flucloxacillin and vancomycin/other	12/71(16.9%)	17/67(25.4%)	1.66 [0.79, 3.47]	
MSSA: other penicillin(s) / cephalosporin(s) / carbapenem(s)	7/30(23.3%)	10/31(32.3%)	1.59 [0.60, 4.18]	
MSSA: vancomycin +/- other	7/56(12.5%)	9/54(16.7%)	1.36 [0.51, 3.65]	
MSSA: other	4/19(21.1%)	1/16(6.3%)	0.32 [0.04, 2.89]	
MRSA	3/21(14.3%)	9/26(34.6%)	2.74 [0.74, 10.15]	
IV CATHETER/IMPLANTED VASCULAR DEVICE-ASSOCIATED?				
No	61/298(20.5%)	51/289(17.6%)	0.90 [0.62, 1.30]	0.34
Yes	9/88(10.2%)	11/80(13.8%)	1.44 [0.60, 3.48]	
ENDOCARDITIS				
No endocarditis	66/370(17.8%)	60/347(17.3%)	1.02 [0.72, 1.45]	0.13
Endocarditis	5/18(27.8%)	2/22(9.1%)	0.30 [0.06, 1.54]	
FOCI OF INFECTION KNOWN				
Foci of infection not known	14/66(21.2%)	17/67(25.4%)	1.31 [0.65, 2.66]	0.30
Foci of infection known	57/322(17.7%)	45/302(14.9%)	0.87 [0.59, 1.29]	
AGE AT LAST BIRTHDAY (YEARS)				
18-54	11/126(8.7%)	8/125(6.4%)	0.76 [0.31, 1.89]	0.57
>54-72	17/126(13.5%)	13/122(10.7%)	0.81 [0.39, 1.67]	
>72	43/136(31.6%)	41/123(33.3%)	1.15 [0.75, 1.77]	

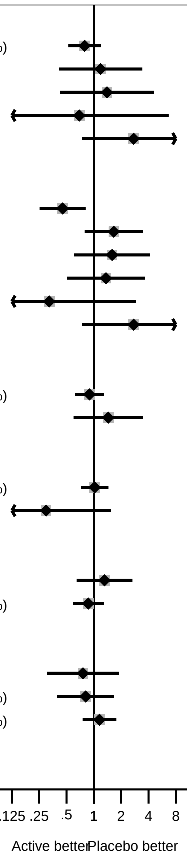

HR [95%CI]

Note: presenting class-level and antibiotic-level categorisation of initial active antibiotics (as per the Statistical Analysis Plan). See **Figure 2(b)** for the three other priority subgroup analyses defined in the Statistical Analysis Plan (time between starting active antibiotics and trial drug, methicillin resistance and foci of infection (deep versus not deep)). All eight priority subgroup analyses were pre-specified in the protocol and the Statistical Analysis Plan.

Figure S2b Twelve other subgroup analyses for bacteriological failure/recurrence or death through 12 weeks (primary endpoint)

	Placebo	Active	Active:placebo HR [95% CI]	Interaction p
INITIAL STUDY DRUG FREQUENCY (PER DAY)				
1	33/175(18.9%)	32/173(18.5%)	1.04 [0.64, 1.70]	0.69
2	36/205(17.6%)	30/191(15.7%)	0.91 [0.56, 1.48]	
INITIAL STUDY DRUG ROUTE				
IV	17/51(33.3%)	17/45(37.8%)	1.29 [0.66, 2.52]	0.37
Oral	52/329(15.8%)	45/319(14.1%)	0.92 [0.62, 1.37]	
GENTAMICIN BETWEEN FIRST BC AND 48 HRS POST-RAND?				
No	49/304(16.1%)	39/286(13.6%)	0.87 [0.57, 1.33]	0.41
Yes	22/84(26.2%)	23/84(27.4%)	1.17 [0.65, 2.09]	
ACTIVE OTHER THAN1ST/GENT BETWEEN1ST+&48H POST-RAND*				
No	10/79(12.7%)	3/66(4.5%)	0.38 [0.10, 1.37]	0.11
Yes	61/309(19.7%)	59/304(19.4%)	1.03 [0.72, 1.48]	
BASELINE CRP (MG/L)				
<=108	18/133(13.5%)	12/119(10.1%)	0.78 [0.38, 1.62]	0.30
>108-184	28/127(22.0%)	22/130(16.9%)	0.75 [0.43, 1.31]	
>184	24/126(19.0%)	27/120(22.5%)	1.33 [0.77, 2.30]	
CHARLSON COMORBIDITY INDEX SCORE*				
0	13/117(11.1%)	6/114(5.3%)	0.48 [0.18, 1.26]	0.07***
1-2	30/143(21.0%)	24/154(15.6%)	0.77 [0.45, 1.31]	
3-4	16/74(21.6%)	15/52(28.8%)	1.47 [0.73, 2.97]	
>=5	12/54(22.2%)	17/49(34.7%)	1.79 [0.86, 3.76]	
TIME FROM RANDOMISATION TO INITIATION OF RANDOMISED TREATMENT (HOURS)*				
0-4	34/180(18.9%)	30/167(18.0%)	0.97 [0.59, 1.59]	1.00
>4-12	23/137(16.8%)	19/127(15.0%)	0.93 [0.51, 1.71]	
>12-24	8/46(17.4%)	9/52(17.3%)	1.09 [0.42, 2.82]	
>24	4/17(23.5%)	4/18(22.2%)	0.97 [0.24, 3.87]	
MODE OF ACQUISITION OF INFECTION*				
Community acquired	45/240(18.8%)	38/245(15.5%)	0.86 [0.56, 1.32]	0.63
Nosocomial infection (onset >=48 hrs after admission)	13/76(17.1%)	12/56(21.4%)	1.30 [0.59, 2.84]	
Healthcare associated (all other)	13/72(18.1%)	12/68(17.6%)	1.06 [0.48, 2.31]	
RANDOMISATION YEAR*				
2012/2013	15/103(14.6%)	21/90(23.3%)	1.77 [0.91, 3.43]	0.15
2014	18/101(17.8%)	12/100(12.0%)	0.66 [0.32, 1.38]	
2015	26/110(23.6%)	19/109(17.4%)	0.75 [0.42, 1.36]	
2016	12/74(16.2%)	10/71(14.1%)	0.96 [0.41, 2.21]	
BASELINE NEUTROPHILS (10^9/L)*				
<=6	20/151(13.2%)	12/135(8.9%)	0.68 [0.33, 1.39]	0.47
>6-9	15/95(15.8%)	15/107(14.0%)	0.92 [0.45, 1.88]	
>9	35/137(25.5%)	34/127(26.8%)	1.15 [0.72, 1.84]	
INITIAL TOTAL DAILY DOSE (MG)**				
600	20/74(27.0%)	15/75(20.0%)	0.80 [0.41, 1.57]	0.47
900	49/306(16.0%)	47/289(16.3%)	1.05 [0.71, 1.57]	
BACTERAEMIC AT RANDOMISATION?**				
Not bacteraemic	46/257(17.9%)	31/228(13.6%)	0.78 [0.50, 1.24]	0.78
Bacteraemic	16/69(23.2%)	17/88(19.3%)	0.88 [0.45, 1.74]	

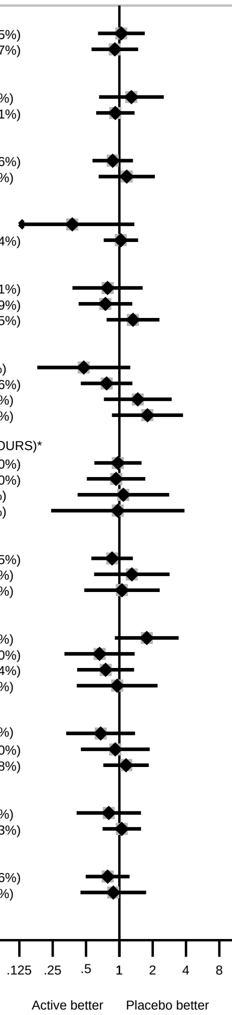

HR [95%CI]

* subgroup analysis pre-specified in the statistical analysis plan but not the protocol
** additional subgroup analysis not in protocol or statistical analysis plan
*** p=0.07 using continuous interactions (splines); p=0.01 using continuous interaction (linear)

Figure S3 Persistence of bacteraemia

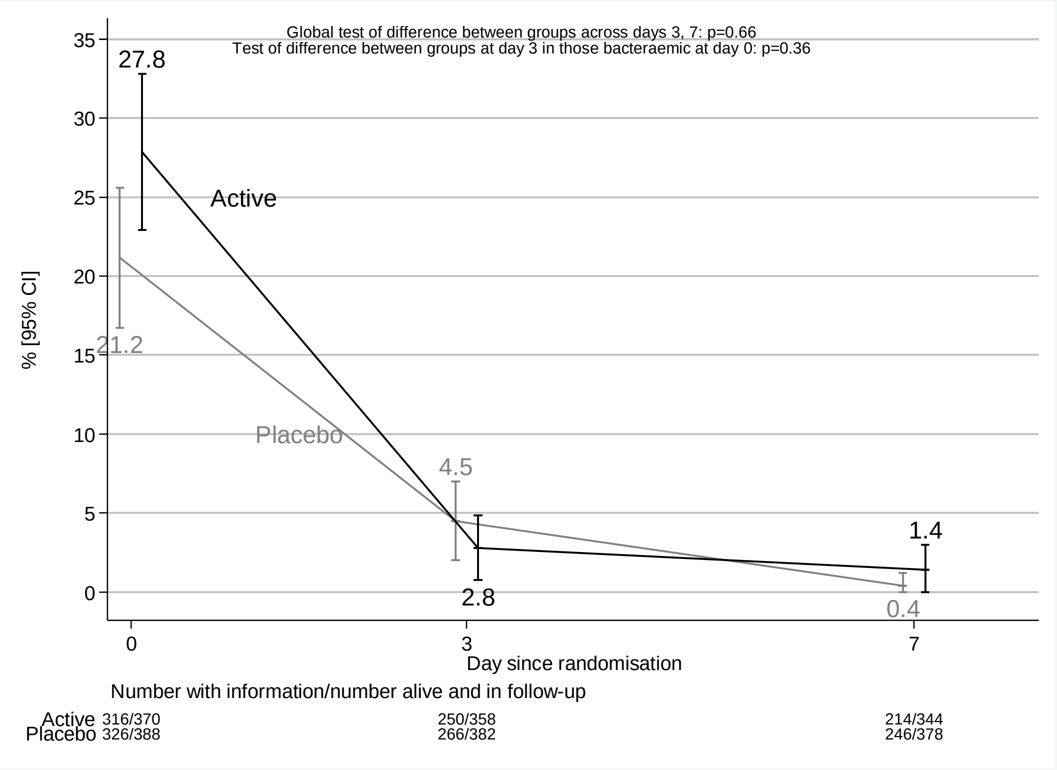

Figure S4 Changes in laboratory test results over 2 weeks from randomisation (a) CRP, (b) ALT, (c) alkaline phosphatase and (d) bilirubin

(a) CRP

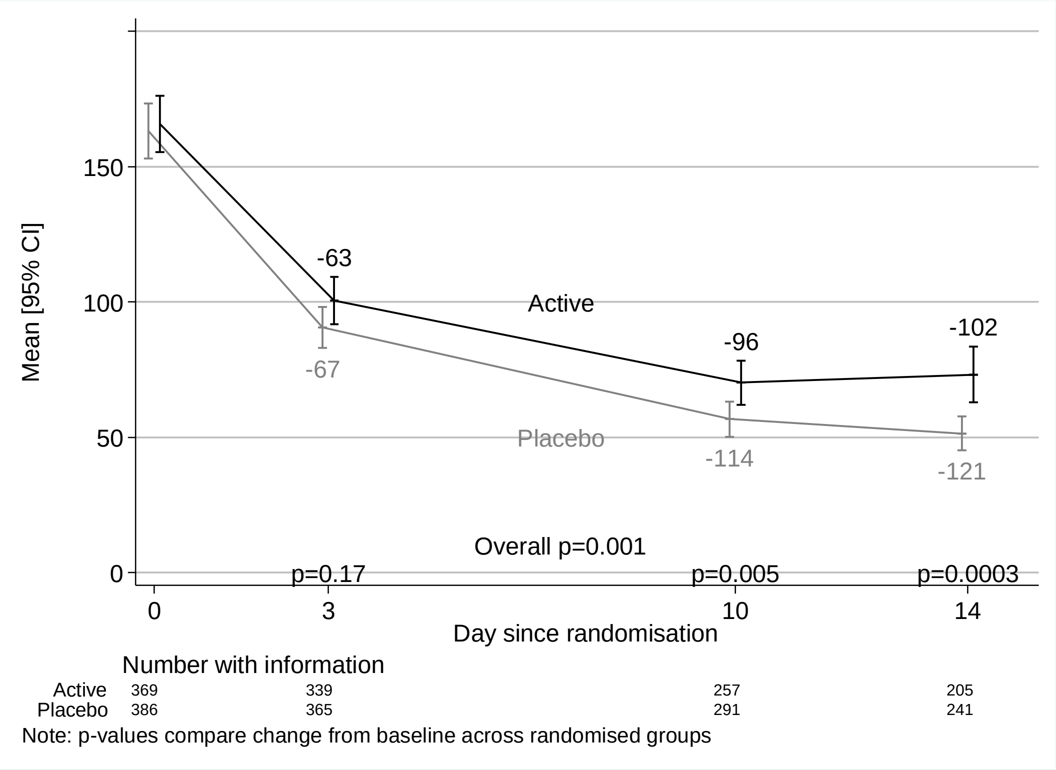

(b) ALT

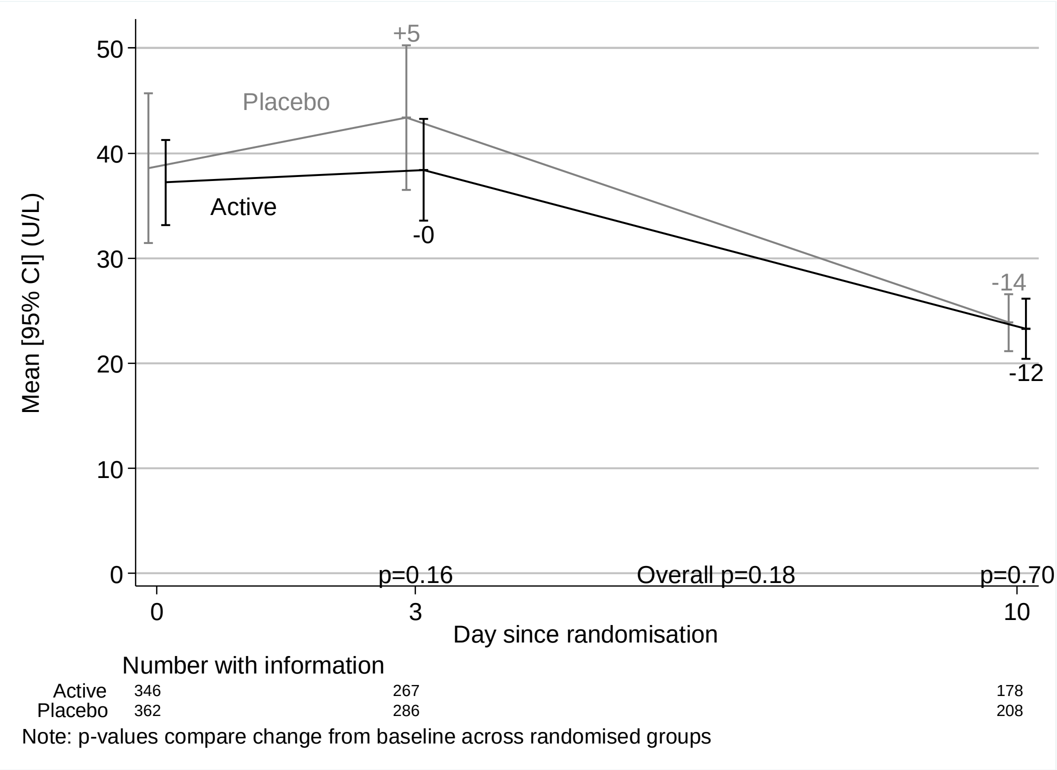

(c) alkaline phosphatase

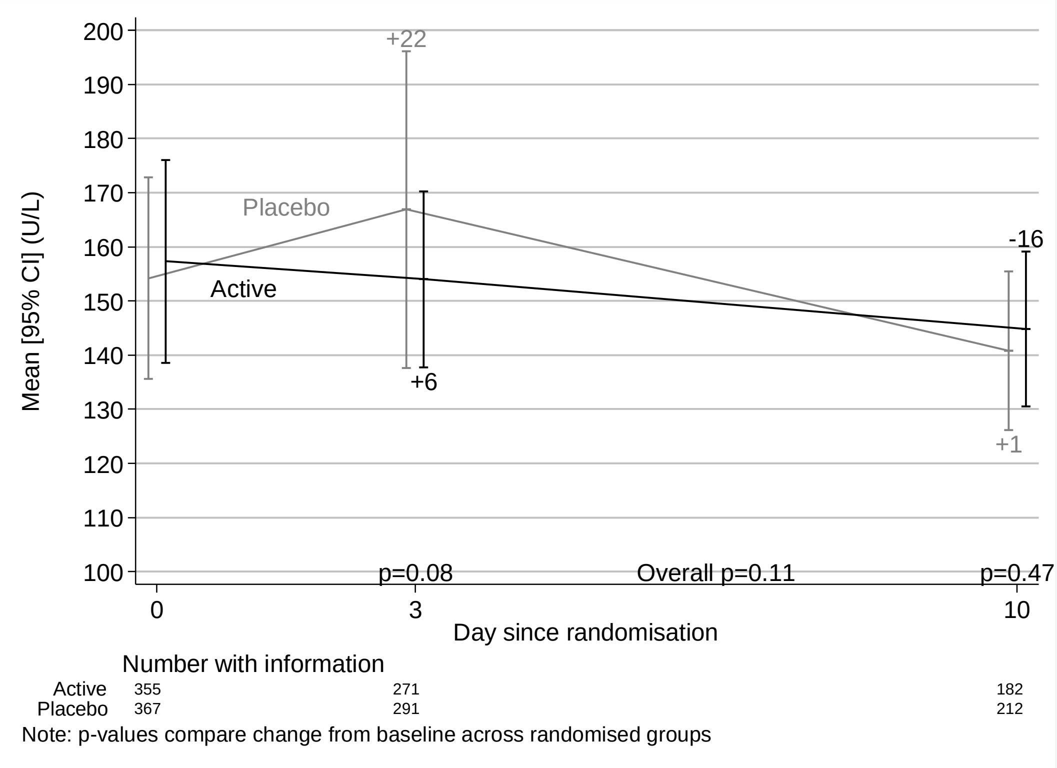

(d) bilirubin

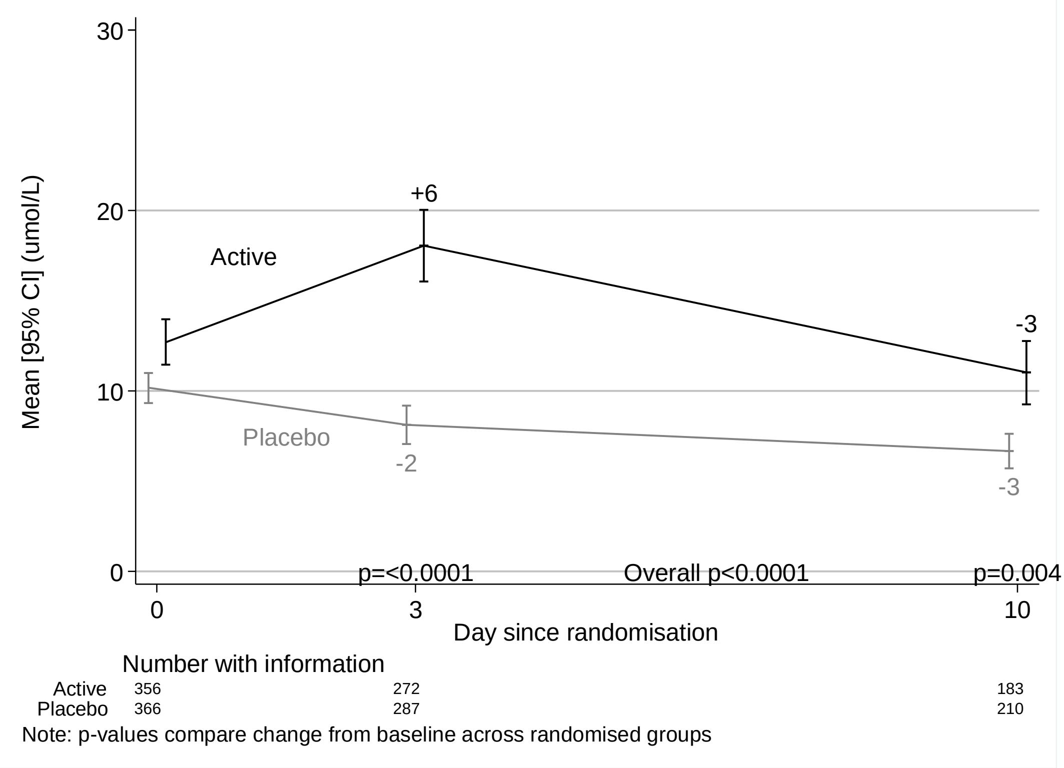

Table S1 Additional characteristics at randomiation

Factor	Placebo N=388*	Rifampicin N=370*	Total N=758*
Likely portal of entry of *S. aureus* into the bloodstream			
Clinically apparent skin or soft tissue infection unrelated to a surgical intervention	131 (33.8%)	124 (33.5%)	255 (33.6%)
Infected surgical wound within last 3 months, with or without associated prosthesis	19 (4.9%)	19 (5.1%)	38 (5.0%)
Peripheral vascular catheter (including arterial line)	23 (5.9%)	26 (7.0%)	49 (6.5%)
Central vascular catheter (including PICC line)	50 (12.9%)	42 (11.4%)	92 (12.1%)
Other implanted vascular device (e.g. pacemaker, stent, graft)	15 (3.9%)	12 (3.2%)	27 (3.6%)
Respiratory	16 (4.1%)	13 (3.5%)	29 (3.8%)
Per-urethral or supra-pubic urinary catheter	7 (1.8%)	8 (2.2%)	15 (2.0%)
Recent (within 1 week of bacteraemia) urological surgery	1 (0.3%)	3 (0.8%)	4 (0.5%)
Not known (absence of any of the above)	110 (28.4%)	108 (29.2%)	218 (28.8%)
Injecting drug user	8 (2.1%)	9 (2.4%)	17 (2.2%)
Corticosteroid Injection Into Joint	4 (1.0%)	2 (0.5%)	6 (0.8%)
Other	2 (0.5%)	3 (0.8%)	5 (0.7%)
Not completed (missing data)	2 (0.5%)	1 (0.3%)	3 (0.4%)
Vascular catheter in situ	102/380 (26.8%)	89/364 (24.5%)	191/744 (25.7%)
Surgery in the last 30 days	53/388 (13.7%)	37/368 (10.1%)	90/756 (11.9%)

Note: showing n(%).

Table S2. Initial infection focus in participants who received open label rifampicin at any point during 12 weeks follow-up

**Infection focus**	Placebo N=52	Rifampicin N=32	Total N=84
Central venous line (including picc line)	1 (1.9%)	2 (6.3%)	3 (3.6%)
Implanted vascular device (e.g. pacemaker, stent, graft)	8 (15.4%)	0 (0.0%)	8 (9.5%)
Infected intravascular thrombus	2 (3.8%)	3 (9.4%)	5 (6.0%)
Native heart valve	6 (11.5%)	2 (6.3%)	8 (9.5%)
Prosthetic heart valve	1 (1.9%)	2 (6.3%)	3 (3.6%)
Native joint	1 (1.9%)	5 (15.6%)	6 (7.1%)
Prosthetic joint	0 (0.0%)	1 (3.1%)	1 (1.2%)
Vertebral bone/disc	13 (25.0%)	8 (25.0%)	21 (25.0%)
Epidural or intraspinal empyema	4 (7.7%)	1 (3.1%)	5 (6.0%)
Deep tissue infection or abscess	6 (11.5%)	3 (9.4%)	9 (10.7%)
Surgical wound	3 (5.8%)	0 (0.0%)	3 (3.6%)
Skin/Soft tissue (excluding wounds)	6 (11.5%)	3 (9.4%)	9 (10.7%)
Pneumonia	2 (3.8%)	1 (3.1%)	3 (3.6%)
Other ‡	6 (11.5%)	0 (0.0%)	6 (7.1%)
Not established	6 (11.5%)	9 (28.1%)	15 (17.9%)

‡ Central nervous system (n=2, both placebo); osteomyelitis (n=1, placebo); Urinary tract (n=3, all placebo)

Table S3 Infection focus management

Factor	Placebo N=388	Rifampicin N=370	Total N=758
Non-device related focus	233	222	455
Drained/removed	39 (16.7%)	36 (16.2%)	75 (16.5%)
Median days from randomisation to drainage/removal (IQR)	4 (2, 11)	4 (2, 8)	4 (2, 10)
Not removed	187 (80.3%)	179 (80.6%)	366 (80.4%)
Not known	7 (3.0%)	7 (3.2%)	14 (3.1%)
Intra-vascular device	88	76	164
Removed	62 (70.5%)	60 (78.9%)	122 (74.4%)
Median days from randomisation to removal (IQR)	-2 (-3, 0)	-1 (-2, 0)	-1 (-2, 0)
Not removed	25 (28.4%)	15 (19.7%)	40 (24.4%)
Not known	1 (1.1%)	1 (1.3%)	2 (1.2%)
Non-vascular prosthetic implant/device	5	9	14
Removed	0 (0.0%)	2 (22.2%)	2 (14.3%)
Median days from randomisation to removal (IQR)	-	7 (2, 11)	7 (2, 11)
Not removed	5 (100.0%)	7 (77.8%)	12 (85.7%)
Any deep-seated focus ‡	159	142	301
Drained/removed	35 (22.0%)	29 (20.4%)	64 (21.3%)
Median days from randomisation to drainage/removal (IQR)	5 (2, 12)	3 (1, 6)	4 (2, 10)
Not removed	118 (74.2%)	109 (76.8%)	227 (75.4%)
Not known	6 (3.8%)	4 (2.8%)	10 (3.3%)

‡ Infection of implanted vascular device, native/prosthetic heart value, native/prosthetic bone/joint, deep tissue infection/abscess (including vertebral bone/disc or other bone infection, epidural or intraspinal empyema, infected intravascular thrombus, brain infection).

Table S4 Primary and secondary causes of deaths through 12 weeks

	Placebo N=56	Rifampicin N=56	Total N=112
**Definitely attributed to *S. aureus***	**16**	**14**	**30**
**Probably attributed to *S. aureus***	**12**	**14**	**26**
**Possibly attributed to *S. aureus***	**4**	**8**	**12**
**Unlikely to be attributed to *S. aureus***	**23**	**18**	**41**
**Attribution to *S. aureus* undeterminable**	**1**	**2**	**3**
**Not attributed to *S. aureus***	**23**	**18**	**41**
**Infections and infestations**	**5**	**4**	**9**
Pneumonia	2	1	3
Pneumonia; Acute myeloid leukaemia, Nasopharyngeal cancer	0	1	1
Pneumonia; Chronic obstructive pulmonary disease	1	0	1
Pneumonia; Renal failure	1	0	1
Urosepsis; Lung disorder	1	0	1
Biliary sepsis	0	1	1
Serratia infection; Pleural infection; Nosocomial infection; Chronic obstructive pulmonary disease	0	1	1
**Cardiac disorders**	**2**	**4**	**6**
Cardiac failure	1	2	3
Cardiac failure; Myocardial infarction	0	1	1
Myocardial ischaemia	1	0	1
Myocardial ischaemia; Renal failure	0	1	1
**Vascular disorders**	**1**	**0**	**1**
Peripheral ischaemia; Colorectal cancer metastatic	1	0	1
**Respiratory, thoracic and mediastinal disorders**	**2**	**2**	**4**
Pneumonia aspiration; Peripheral vascular disorder	0	1	1
Pulmonary embolism; Colon cancer metastatic	0	1	1
Pulmonary embolism; Death	1	0	1
Pulmonary oedema; Peripheral vascular disorder	1	0	1
**Gastrointestinal disorders**	**2**	**3**	**5**
Diarrhoea	1	0	1
Gastrointestinal haemorrhage; Angiodysplasia	0	1	1
Pancreatitis; Intestinal perforation	1	0	1
Pancreatitis acute; End stage renal disease	0	1	1
Upper gastrointestinal haemorrhage	0	1	1
**Neoplasms benign, malignant and unspecified (incl cysts and polyps)**	**5**	**5**	**10**
Chronic myeloid leukaemia	1	1	2
Lymphoma; Cardiac failure, Diabetes mellitus	0	1	1
Plasma cell myeloma; Endocarditis	1	0	1
Prostate cancer metastatic	0	1	1
Lung cancer metastatic	0	1	1
Renal cancer metastatic	1	0	1
Breast cancer metastatic	1	1	2
Prostate cancer	1	0	1
**General disorders and administration site conditions**	**3**	**0**	**3**
Death; Cerebrovascular disorder	1	0	1
Death; Dementia	1	0	1
Sudden death	1	0	1
**Nervous system disorders**	**3**	**0**	**3**
Cerebral haemorrhage	1	0	1
Cerebrovascular accident	1	0	1
Dementia Alzheimer's type	1	0	1

	Placebo N=56	Rifampicin N=56	Total N=112
**Attributed to *S. aureus***	**32**	**36**	**68**
**Infections and infestations**	**29**	**28**	**57**
Endocarditis; Staphylococcal bacteraemia	2	1	3
Osteomyelitis; Staphylococcal bacteraemia	0	1	1
Pneumonia	0	1	1
Pneumonia; Decubitus ulcer, Acute myocardial infarction	0	1	1
Pneumonia; Staphylococcal bacteraemia	0	3	7
Pneumonia; Staphylococcal bacteraemia, Chronic obstructive pulmonary disease	0	1	1
Sepsis	0	4	4
Sepsis; Renal failure, Chronic obstructive pulmonary disease	1	0	1
Sepsis; Staphylococcal bacteraemia	0	1	1
Staphylococcal bacteraemia	3	2	5
Staphylococcal bacteraemia; Alcoholism, Diabetes mellitus	1	0	1
Staphylococcal bacteraemia; Bone cancer metastatic, Colorectal cancer metastatic	1	0	1
Staphylococcal bacteraemia; Brain injury	1	0	1
Staphylococcal bacteraemia; Carcinoid tumour	0	1	1
Staphylococcal bacteraemia; Death	1	0	1
Staphylococcal bacteraemia; Dementia	0	1	1
Staphylococcal bacteraemia; Device related infection, Endocarditis	0	1	1
Staphylococcal bacteraemia; Endocarditis	4	2	6
Staphylococcal bacteraemia; Endocarditis, Atrioventricular block complete	0	1	1
Staphylococcal bacteraemia; Endocarditis, Gastrointestinal carcinoma	1	0	1
Staphylococcal bacteraemia; Graft infection, Endocarditis	0	1	1
Staphylococcal bacteraemia; Intervertebral discitis	0	1	1
Staphylococcal bacteraemia; Lung cancer metastatic	1	0	1
Staphylococcal bacteraemia; Lung neoplasm malignant	0	1	1
Staphylococcal bacteraemia; Mediastinal abscess, Coronary artery bypass	1	0	1
Staphylococcal bacteraemia; Parotitis, Prostate cancer metastatic	0	1	1
Staphylococcal bacteraemia; Peripheral artery aneurysm	1	0	1
Staphylococcal bacteraemia; Pneumonia	1	1	2
Staphylococcal bacteraemia; Pneumonia aspiration	0	1	1
Staphylococcal bacteraemia; Psoas abscess	1	0	1
Staphylococcal bacteraemia; Pyelonephritis	1	0	1
Staphylococcal bacteraemia; Pyomyositis, Extradural abscess	1	0	1
Staphylococcal bacteraemia; Renal failure, Extradural abscess	1	0	1
Staphylococcal bacteraemia; Soft tissue infection	1	0	1
Staphylococcal bacteraemia; Tongue neoplasm malignant stage unspecified, Alcohol abuse	0	1	1
Arthritis bacterial; Osteomyelitis, Staphylococcal bacteraemia	1	0	1
**Cardiac disorders**	**0**	**2**	**2**
Cardiac failure; Endocarditis, Staphylococcal bacteraemia	0	1	1
Cardiopulmonary failure	0	1	1
**Respiratory, thoracic and mediastinal disorders**	**0**	**1**	**1**
Pneumothorax; Pneumonia, Staphylococcal bacteraemia	0	1	1
**Gastrointestinal disorders**	**0**	**2**	**2**
Intestinal ischaemia; Staphylococcal bacteraemia	0	1	1
Intestinal ischaemia; Staphylococcal bacteraemia, Endocarditis	0	1	1
**Renal and urinary disorders**	**3**	**0**	**3**
Renal failure; Spinal cord compression, Staphylococcal bacteraemia	1	0	1
Renal tubular acidosis; Staphylococcal bacteraemia	1	0	1
Acute kidney injury; Pseudomonal bacteraemia	1	0	1
**Neoplasms benign, malignant and unspecified (incl cysts and polyps)**	**0**	**3**	**3**

	Placebo N=56	Rifampicin N=56	Total N=112
Mycosis fungoides; Staphylococcal bacteraemia	0	1	1
Lung cancer metastatic	0	1	1
Metastatic carcinoma of the bladder	0	1	1
**Attribution to *S. aureus* undeterminable**	**1**	**2**	**3**
**Infections and infestations**	**0**	**1**	**1**
Pneumonia; Chronic obstructive pulmonary disease	0	1	1
**Cardiac disorders**	**1**	**0**	**1**
Left ventricular failure; Lung disorder, Sepsis	1	0	1
**General disorders and administration site conditions**	**0**	**1**	**1**
Death	0	1	1

Note: Where secondary cause(s) of death were adjudicated by the independent review committee, these are provided after the primary cause of death

Table S5 Summary of SAEs, Grade 3/4 and antibiotic-modifying adverse events

Outcome	Placebo N=388	Rifampicin N=370	Total N=758	p*
**SAEs**				
**Any**	**94 (24.2%) 116**	**101 (27.3%) 112**	**195 (25.7%) 228**	**0.36**
Infections and infestations	39 (10.1%) 40	37 (10.0%) 38	76 (10.0%) 78	1.00
Cardiac disorders	13 (3.4%) 15	5 (1.4%) 6	18 (2.4%) 21	0.09
Vascular disorders	2 (0.5%) 2	4 (1.1%) 4	6 (0.8%) 6	0.44
Respiratory, thoracic and mediastinal disorders	12 (3.1%) 12	6 (1.6%) 6	18 (2.4%) 18	0.23
Gastrointestinal disorders	7 (1.8%) 7	10 (2.7%) 12	17 (2.2%) 19	0.47
Hepatobiliary disorders	0 (0.0%) 0	2 (0.5%) 2	2 (0.3%) 2	0.24
Skin and subcutaneous tissue disorders	1 (0.3%) 1	1 (0.3%) 1	2 (0.3%) 2	1.00
Renal and urinary disorders	4 (1.0%) 4	10 (2.7%) 10	14 (1.8%) 14	0.11
Neoplasms benign, malignant and unspecified (incl cysts and polyps)	7 (1.8%) 7	11 (3.0%) 12	18 (2.4%) 19	0.34
Congenital, familial and genetic disorders	1 (0.3%) 1	0 (0.0%) 0	1 (0.1%) 1	1.00
General disorders and administration site conditions	12 (3.1%) 12	11 (3.0%) 11	23 (3.0%) 23	1.00
Investigations	0 (0.0%) 0	1 (0.3%) 1	1 (0.1%) 1	0.49
Injury, poisoning and procedural complications	5 (1.3%) 5	3 (0.8%) 3	8 (1.1%) 8	0.73
Blood and lymphatic system disorders	1 (0.3%) 1	1 (0.3%) 1	2 (0.3%) 2	1.00
Metabolism and nutrition disorders	1 (0.3%) 1	3 (0.8%) 3	4 (0.5%) 4	0.36
Psychiatric disorders	2 (0.5%) 2	0 (0.0%) 0	2 (0.3%) 2	0.50
Nervous system disorders	5 (1.3%) 6	2 (0.5%) 2	7 (0.9%) 8	0.45
**Grade 3/4 adverse events**				
**Any**	**131 (33.8%) 193**	**129 (34.9%) 209**	**260 (34.3%) 402**	**0.76**
Infections and infestations	45 (11.6%) 53	40 (10.8%) 48	85 (11.2%) 101	0.82
Cardiac disorders	15 (3.9%) 17	6 (1.6%) 8	21 (2.8%) 25	0.08
Vascular disorders	7 (1.8%) 7	5 (1.4%) 5	12 (1.6%) 12	0.77
Respiratory, thoracic and mediastinal disorders	16 (4.1%) 17	10 (2.7%) 11	26 (3.4%) 28	0.32
Gastrointestinal disorders	21 (5.4%) 24	29 (7.8%) 40	50 (6.6%) 64	0.19
Hepatobiliary disorders	0 (0.0%) 0	3 (0.8%) 3	3 (0.4%) 3	0.12
Skin and subcutaneous tissue disorders	7 (1.8%) 7	5 (1.4%) 5	12 (1.6%) 12	0.77
Musculoskeletal and connective tissue disorders	2 (0.5%) 2	0 (0.0%) 0	2 (0.3%) 2	0.50
Renal and urinary disorders	9 (2.3%) 9	19 (5.1%) 20	28 (3.7%) 29	0.053
Neoplasms benign, malignant and unspecified (incl cysts and polyps)	7 (1.8%) 7	11 (3.0%) 12	18 (2.4%) 19	0.34
Reproductive system and breast disorders	0 (0.0%) 0	1 (0.3%) 1	1 (0.1%) 1	0.49
Congenital, familial and genetic disorders	1 (0.3%) 1	0 (0.0%) 0	1 (0.1%) 1	1.00
General disorders and administration site conditions	11 (2.8%) 11	12 (3.2%) 12	23 (3.0%) 23	0.83
Investigations	6 (1.5%) 6	11 (3.0%) 16	17 (2.2%) 22	0.22
Injury, poisoning and procedural complications	6 (1.5%) 6	5 (1.4%) 5	11 (1.5%) 11	1.00
Surgical and medical procedures	0 (0.0%) 0	1 (0.3%) 1	1 (0.1%) 1	0.49
Blood and lymphatic system disorders	3 (0.8%) 3	5 (1.4%) 6	8 (1.1%) 9	0.50
Metabolism and nutrition disorders	3 (0.8%) 3	5 (1.4%) 6	8 (1.1%) 9	0.50
Psychiatric disorders	5 (1.3%) 5	5 (1.4%) 6	10 (1.3%) 11	1.00
Nervous system disorders	11 (2.8%) 14	4 (1.1%) 4	15 (2.0%) 18	0.12
Eye disorders	1 (0.3%) 1	0 (0.0%) 0	1 (0.1%) 1	1.00
**Antibiotic-modifying adverse events**				
**Any**	**39 (10.1%) 52**	**63 (17.0%) 89**	**102 (13.5%) 141**	**0.006**
Infections and infestations	3 (0.8%) 3	5 (1.4%) 5	8 (1.1%) 8	0.50
Respiratory, thoracic and mediastinal disorders	2 (0.5%) 4	0 (0.0%) 0	2 (0.3%) 4	0.50
Gastrointestinal disorders	8 (2.1%) 9	24 (6.5%) 32	32 (4.2%) 41	0.003
Hepatobiliary disorders	0 (0.0%) 0	2 (0.5%) 2	2 (0.3%) 2	0.24
Skin and subcutaneous tissue disorders	7 (1.8%) 9	8 (2.2%) 9	15 (2.0%) 18	0.80
Renal and urinary disorders	1 (0.3%) 2	8 (2.2%) 10	9 (1.2%) 12	0.02
General disorders and administration site conditions	4 (1.0%) 4	13 (3.5%) 13	17 (2.2%) 17	0.03
Investigations	12 (3.1%) 13	12 (3.2%) 14	24 (3.2%) 27	1.00
Injury, poisoning and procedural complications	1 (0.3%) 1	0 (0.0%) 0	1 (0.1%) 1	1.00
Blood and lymphatic system disorders	1 (0.3%) 1	3 (0.8%) 3	4 (0.5%) 4	0.36
Metabolism and nutrition disorders	2 (0.5%) 3	0 (0.0%) 0	2 (0.3%) 3	0.50
Psychiatric disorders	1 (0.3%) 2	0 (0.0%) 0	1 (0.1%) 2	1.00
Nervous system disorders	1 (0.3%) 1	1 (0.3%) 1	2 (0.3%) 2	1.00

Note: Showing number of patients with one or more event (% of participants) number of events (e.g., '2 (20.0%) 3,' would indicate a total of 3 events in a total of 2 participants)

* Fisher's exact test

Table S6 Serious adverse events

	Placebo N=388	Rifampicin N=370	Total N=758	p*
**MedDRA code**				
**Any**	**94 (24.2%) 116**	**101 (27.3%) 112**	**195 (25.7%) 228**	**0.36**
**Infections and infestations**	**39 (10.1%) 40**	**37 (10.0%) 38**	**76 (10.0%) 78**	**1.00**
Cellulitis	1 (0.3%) 1	0 (0.0%) 0	1 (0.1%) 1	
Clostridium difficile colitis	1 (0.3%) 1	0 (0.0%) 0	1 (0.1%) 1	
Diarrhoea infectious	1 (0.3%) 1	0 (0.0%) 0	1 (0.1%) 1	
Endocarditis	3 (0.8%) 3	2 (0.5%) 2	5 (0.7%) 5	
Endocarditis, Graft infection	0 (0.0%) 0	1 (0.3%) 1	1 (0.1%) 1	
Gastroenteritis	1 (0.3%) 1	0 (0.0%) 0	1 (0.1%) 1	
Infection	0 (0.0%) 0	1 (0.3%) 1	1 (0.1%) 1	
Lower respiratory tract infection	1 (0.3%) 1	0 (0.0%) 0	1 (0.1%) 1	
Pneumonia	12 (3.1%) 12	10 (2.7%) 10	22 (2.9%) 22	
Pneumonia, Osteomyelitis	0 (0.0%) 0	1 (0.3%) 1	1 (0.1%) 1	
Pneumonia, Sepsis	1 (0.3%) 1	1 (0.3%) 1	2 (0.3%) 2	
Pneumonia, Staphylococcal bacteraemia	0 (0.0%) 0	1 (0.3%) 1	1 (0.1%) 1	
Pneumonia, Staphylococcal bacteraemia, Confusional state, Cellulitis	0 (0.0%) 0	1 (0.3%) 1	1 (0.1%) 1	
Postoperative wound infection	1 (0.3%) 1	0 (0.0%) 0	1 (0.1%) 1	
Pyelonephritis	1 (0.3%) 1	0 (0.0%) 0	1 (0.1%) 1	
Sepsis	3 (0.8%) 3	4 (1.1%) 4	7 (0.9%) 7	
Septic shock, Necrotising fasciitis	1 (0.3%) 1	0 (0.0%) 0	1 (0.1%) 1	
Subcutaneous abscess	1 (0.3%) 1	0 (0.0%) 0	1 (0.1%) 1	
Urinary tract infection	2 (0.5%) 2	2 (0.5%) 2	4 (0.5%) 4	
Urosepsis	1 (0.3%) 1	0 (0.0%) 0	1 (0.1%) 1	
Groin abscess	1 (0.3%) 1	0 (0.0%) 0	1 (0.1%) 1	
Abscess limb	0 (0.0%) 0	1 (0.3%) 1	1 (0.1%) 1	
Staphylococcal bacteraemia	1 (0.3%) 1	1 (0.3%) 1	2 (0.3%) 2	
Staphylococcal bacteraemia, Cardiac tamponade	0 (0.0%) 0	1 (0.3%) 1	1 (0.1%) 1	
Arthritis bacterial	1 (0.3%) 1	0 (0.0%) 0	1 (0.1%) 1	
Arthritis bacterial, Pneumonia, Intervertebral discitis, Staphylococcal bacteraemia	1 (0.3%) 1	0 (0.0%) 0	1 (0.1%) 1	
Escherichia bacteraemia	0 (0.0%) 0	1 (0.3%) 1	1 (0.1%) 1	
Staphylococcal sepsis	3 (0.8%) 3	2 (0.5%) 2	5 (0.7%) 5	
Staphylococcal sepsis, Tongue neoplasm	0 (0.0%) 0	1 (0.3%) 1	1 (0.1%) 1	

	Placebo N=388	Rifampicin N=370	Total N=758	p*
Biliary sepsis	0 (0.0%) 0	1 (0.3%) 1	1 (0.1%) 1	
Intervertebral discitis	1 (0.3%) 1	0 (0.0%) 0	1 (0.1%) 1	
Pseudomonas infection	0 (0.0%) 0	1 (0.3%) 1	1 (0.1%) 1	
Extradural abscess	0 (0.0%) 0	1 (0.3%) 1	1 (0.1%) 1	
Device related infection	1 (0.3%) 1	1 (0.3%) 1	2 (0.3%) 2	
Staphylococcal parotitis	0 (0.0%) 0	1 (0.3%) 1	1 (0.1%) 1	
Candida infection	0 (0.0%) 0	1 (0.3%) 1	1 (0.1%) 1	
Disseminated varicella zoster vaccine virus infection	0 (0.0%) 0	1 (0.3%) 1	1 (0.1%) 1	
**Cardiac disorders**	**13 (3.4%) 15**	**5 (1.4%) 6**	**18 (2.4%) 21**	**0.09**
Atrial fibrillation, Acute kidney injury, Thrombocytopenia	0 (0.0%) 0	1 (0.3%) 1	1 (0.1%) 1	
Atrioventricular block complete	0 (0.0%) 0	1 (0.3%) 1	1 (0.1%) 1	
Cardiac arrest	5 (1.3%) 7	1 (0.3%) 1	6 (0.8%) 8	
Cardiac arrest, Pulmonary embolism	1 (0.3%) 1	0 (0.0%) 0	1 (0.1%) 1	
Cardiac failure	1 (0.3%) 1	0 (0.0%) 0	1 (0.1%) 1	
Cardiac failure congestive	1 (0.3%) 1	0 (0.0%) 0	1 (0.1%) 1	
Cardiac tamponade	1 (0.3%) 1	0 (0.0%) 0	1 (0.1%) 1	
Cardio-respiratory arrest	0 (0.0%) 0	1 (0.3%) 1	1 (0.1%) 1	
Myocardial infarction	1 (0.3%) 1	0 (0.0%) 0	1 (0.1%) 1	
Myocardial ischaemia	1 (0.3%) 1	0 (0.0%) 0	1 (0.1%) 1	
Tachycardia	1 (0.3%) 1	0 (0.0%) 0	1 (0.1%) 1	
Ventricular tachycardia	0 (0.0%) 0	1 (0.3%) 2	1 (0.1%) 2	
Ischaemic cardiomyopathy	1 (0.3%) 1	0 (0.0%) 0	1 (0.1%) 1	
**Vascular disorders**	**2 (0.5%) 2**	**4 (1.1%) 4**	**6 (0.8%) 6**	**0.44**
Hypotension	0 (0.0%) 0	1 (0.3%) 1	1 (0.1%) 1	
Peripheral ischaemia	2 (0.5%) 2	1 (0.3%) 1	3 (0.4%) 3	
Deep vein thrombosis	0 (0.0%) 0	2 (0.5%) 2	2 (0.3%) 2	
**Respiratory, thoracic and mediastinal disorders**	**12 (3.1%) 12**	**6 (1.6%) 6**	**18 (2.4%) 18**	**0.23**
Asthma	1 (0.3%) 1	0 (0.0%) 0	1 (0.1%) 1	
Chronic obstructive pulmonary disease	2 (0.5%) 2	0 (0.0%) 0	2 (0.3%) 2	
Dyspnoea	0 (0.0%) 0	1 (0.3%) 1	1 (0.1%) 1	
Dyspnoea, Pulmonary oedema	0 (0.0%) 0	1 (0.3%) 1	1 (0.1%) 1	
Epistaxis	1 (0.3%) 1	0 (0.0%) 0	1 (0.1%) 1	
Hypoxia	1 (0.3%) 1	0 (0.0%) 0	1 (0.1%) 1	

	Placebo N=388	Rifampicin N=370	Total N=758	p*
Pleural effusion	1 (0.3%) 1	0 (0.0%) 0	1 (0.1%) 1	
Pneumonia aspiration	2 (0.5%) 2	1 (0.3%) 1	3 (0.4%) 3	
Pulmonary embolism	1 (0.3%) 1	0 (0.0%) 0	1 (0.1%) 1	
Pulmonary oedema	2 (0.5%) 2	1 (0.3%) 1	3 (0.4%) 3	
Pulmonary oedema, Acute kidney injury, Staphylococcal bacteraemia	0 (0.0%) 0	1 (0.3%) 1	1 (0.1%) 1	
Respiratory arrest	1 (0.3%) 1	0 (0.0%) 0	1 (0.1%) 1	
Respiratory failure	0 (0.0%) 0	1 (0.3%) 1	1 (0.1%) 1	
**Gastrointestinal disorders**	**7 (1.8%) 7**	**10 (2.7%) 12**	**17 (2.2%) 19**	**0.47**
Colitis ulcerative	0 (0.0%) 0	1 (0.3%) 1	1 (0.1%) 1	
Diarrhoea	0 (0.0%) 0	1 (0.3%) 1	1 (0.1%) 1	
Diarrhoea, Vomiting	0 (0.0%) 0	1 (0.3%) 1	1 (0.1%) 1	
Dyspepsia	1 (0.3%) 1	0 (0.0%) 0	1 (0.1%) 1	
Gastrointestinal angiodysplasia	0 (0.0%) 0	1 (0.3%) 1	1 (0.1%) 1	
Gastrointestinal haemorrhage	1 (0.3%) 1	0 (0.0%) 0	1 (0.1%) 1	
Intestinal ischaemia	0 (0.0%) 0	2 (0.5%) 2	2 (0.3%) 2	
Intestinal obstruction	0 (0.0%) 0	1 (0.3%) 1	1 (0.1%) 1	
Nausea	0 (0.0%) 0	1 (0.3%) 1	1 (0.1%) 1	
Pancreatitis	2 (0.5%) 2	0 (0.0%) 0	2 (0.3%) 2	
Pancreatitis acute	0 (0.0%) 0	1 (0.3%) 1	1 (0.1%) 1	
Upper gastrointestinal haemorrhage	0 (0.0%) 0	1 (0.3%) 1	1 (0.1%) 1	
Vomiting	1 (0.3%) 1	2 (0.5%) 2	3 (0.4%) 3	
Lower gastrointestinal haemorrhage	2 (0.5%) 2	0 (0.0%) 0	2 (0.3%) 2	
**Hepatobiliary disorders**	**0 (0.0%) 0**	**2 (0.5%) 2**	**2 (0.3%) 2**	**0.24**
Hepatic failure	0 (0.0%) 0	2 (0.5%) 2	2 (0.3%) 2	
**Skin and subcutaneous tissue disorders**	**1 (0.3%) 1**	**1 (0.3%) 1**	**2 (0.3%) 2**	**1.00**
Erythema	0 (0.0%) 0	1 (0.3%) 1	1 (0.1%) 1	
Rash vesicular	1 (0.3%) 1	0 (0.0%) 0	1 (0.1%) 1	
**Renal and urinary disorders**	**4 (1.0%) 4**	**10 (2.7%) 10**	**14 (1.8%) 14**	**0.11**
Renal tubular acidosis	1 (0.3%) 1	0 (0.0%) 0	1 (0.1%) 1	
Renal impairment	0 (0.0%) 0	1 (0.3%) 1	1 (0.1%) 1	
Acute kidney injury	0 (0.0%) 0	7 (1.9%) 7	7 (0.9%) 7	
Acute kidney injury, Hypernatraemia	0 (0.0%) 0	1 (0.3%) 1	1 (0.1%) 1	
Acute kidney injury, Respiratory failure	1 (0.3%) 1	0 (0.0%) 0	1 (0.1%) 1	

	Placebo N=388	Rifampicin N=370	Total N=758	p*
Acute kidney injury, Sepsis	1 (0.3%) 1	0 (0.0%) 0	1 (0.1%) 1	
Acute kidney injury, Urinary retention	0 (0.0%) 0	1 (0.3%) 1	1 (0.1%) 1	
Acute kidney injury, Vomiting	1 (0.3%) 1	0 (0.0%) 0	1 (0.1%) 1	
**Neoplasms benign, malignant and unspecified (incl cysts and polyps)**	**7 (1.8%) 7**	**11 (3.0%) 12**	**18 (2.4%) 19**	**0.34**
Bladder cancer	0 (0.0%) 0	1 (0.3%) 1	1 (0.1%) 1	
Breast cancer	0 (0.0%) 0	1 (0.3%) 1	1 (0.1%) 1	
Chronic lymphocytic leukaemia	0 (0.0%) 0	2 (0.5%) 2	2 (0.3%) 2	
Chronic myeloid leukaemia	1 (0.3%) 1	0 (0.0%) 0	1 (0.1%) 1	
Colon cancer	1 (0.3%) 1	0 (0.0%) 0	1 (0.1%) 1	
Lung adenocarcinoma	0 (0.0%) 0	1 (0.3%) 1	1 (0.1%) 1	
Mycosis fungoides	0 (0.0%) 0	1 (0.3%) 1	1 (0.1%) 1	
Prostate cancer metastatic	1 (0.3%) 1	1 (0.3%) 1	2 (0.3%) 2	
Lung cancer metastatic	1 (0.3%) 1	2 (0.5%) 2	3 (0.4%) 3	
Colorectal cancer metastatic	1 (0.3%) 1	1 (0.3%) 1	2 (0.3%) 2	
Breast cancer metastatic	1 (0.3%) 1	0 (0.0%) 0	1 (0.1%) 1	
Lung neoplasm malignant	0 (0.0%) 0	1 (0.3%) 1	1 (0.1%) 1	
Renal cell carcinoma	1 (0.3%) 1	0 (0.0%) 0	1 (0.1%) 1	
Hepatocellular carcinoma	0 (0.0%) 0	1 (0.3%) 1	1 (0.1%) 1	
**Congenital, familial and genetic disorders**	**1 (0.3%) 1**	**0 (0.0%) 0**	**1 (0.1%) 1**	**1.00**
Sickle cell anaemia, Osteomyelitis	1 (0.3%) 1	0 (0.0%) 0	1 (0.1%) 1	
**General disorders and administration site conditions**	**12 (3.1%) 12**	**11 (3.0%) 11**	**23 (3.0%) 23**	**1.00**
Asthenia	2 (0.5%) 2	2 (0.5%) 2	4 (0.5%) 4	
Chest pain	2 (0.5%) 2	1 (0.3%) 1	3 (0.4%) 3	
Death	0 (0.0%) 0	1 (0.3%) 1	1 (0.1%) 1	
Drug withdrawal syndrome	0 (0.0%) 0	1 (0.3%) 1	1 (0.1%) 1	
Pain	0 (0.0%) 0	1 (0.3%) 1	1 (0.1%) 1	
Pyrexia	0 (0.0%) 0	2 (0.5%) 2	2 (0.3%) 2	
Pyrexia, Urinary tract infection	1 (0.3%) 1	0 (0.0%) 0	1 (0.1%) 1	
General physical health deterioration	3 (0.8%) 3	0 (0.0%) 0	3 (0.4%) 3	
General physical health deterioration, Urinary tract infection	0 (0.0%) 0	1 (0.3%) 1	1 (0.1%) 1	
Multiple organ dysfunction syndrome	3 (0.8%) 3	1 (0.3%) 1	4 (0.5%) 4	
Multiple organ dysfunction syndrome, Peritonitis	1 (0.3%) 1	0 (0.0%) 0	1 (0.1%) 1	
Multiple organ dysfunction syndrome, Sepsis	0 (0.0%) 0	1 (0.3%) 1	1 (0.1%) 1	

	Placebo N=388	Rifampicin N=370	Total N=758	p*
**Investigations**	**0 (0.0%) 0**	**1 (0.3%) 1**	**1 (0.1%) 1**	**0.49**
Alanine aminotransferase increased	0 (0.0%) 0	1 (0.3%) 1	1 (0.1%) 1	
**Injury, poisoning and procedural complications**	**5 (1.3%) 5**	**3 (0.8%) 3**	**8 (1.1%) 8**	**0.73**
Fall	0 (0.0%) 0	1 (0.3%) 1	1 (0.1%) 1	
Femoral neck fracture	1 (0.3%) 1	1 (0.3%) 1	2 (0.3%) 2	
Subdural haematoma	0 (0.0%) 0	1 (0.3%) 1	1 (0.1%) 1	
Vascular pseudoaneurysm	1 (0.3%) 1	0 (0.0%) 0	1 (0.1%) 1	
Crush injury	1 (0.3%) 1	0 (0.0%) 0	1 (0.1%) 1	
Toxicity to various agents	1 (0.3%) 1	0 (0.0%) 0	1 (0.1%) 1	
Stoma obstruction	1 (0.3%) 1	0 (0.0%) 0	1 (0.1%) 1	
**Blood and lymphatic system disorders**	**1 (0.3%) 1**	**1 (0.3%) 1**	**2 (0.3%) 2**	**1.00**
Disseminated intravascular coagulation	0 (0.0%) 0	1 (0.3%) 1	1 (0.1%) 1	
Sickle cell anaemia with crisis	1 (0.3%) 1	0 (0.0%) 0	1 (0.1%) 1	
**Metabolism and nutrition disorders**	**1 (0.3%) 1**	**3 (0.8%) 3**	**4 (0.5%) 4**	**0.36**
Diabetes mellitus	0 (0.0%) 0	1 (0.3%) 1	1 (0.1%) 1	
Diabetic ketoacidosis	0 (0.0%) 0	1 (0.3%) 1	1 (0.1%) 1	
Hyperglycaemia	1 (0.3%) 1	1 (0.3%) 1	2 (0.3%) 2	
**Psychiatric disorders**	**2 (0.5%) 2**	**0 (0.0%) 0**	**2 (0.3%) 2**	**0.50**
Agitation	1 (0.3%) 1	0 (0.0%) 0	1 (0.1%) 1	
Confusional state	1 (0.3%) 1	0 (0.0%) 0	1 (0.1%) 1	
**Nervous system disorders**	**5 (1.3%) 6**	**2 (0.5%) 2**	**7 (0.9%) 8**	**0.45**
Cerebral haemorrhage	1 (0.3%) 1	0 (0.0%) 0	1 (0.1%) 1	
Cerebrovascular accident	1 (0.3%) 1	0 (0.0%) 0	1 (0.1%) 1	
Generalised tonic-clonic seizure	0 (0.0%) 0	1 (0.3%) 1	1 (0.1%) 1	
Seizure	2 (0.5%) 2	0 (0.0%) 0	2 (0.3%) 2	
Syncope	1 (0.3%) 1	0 (0.0%) 0	1 (0.1%) 1	
Alcoholic seizure	1 (0.3%) 1	0 (0.0%) 0	1 (0.1%) 1	
Neurological symptom	0 (0.0%) 0	1 (0.3%) 1	1 (0.1%) 1	

Note: Table shows number of participants with one or more episode (% of participants) [number of episodes] (e.g., '2 (20.0%) [3],' would indicate a total of 3 episodes in 2 participants)

* Fisher’s exact test

Table S7 Drug-modifying adverse events

	Placebo N=388	Rifampicin N=370	Total N=758	p*
**MedDRA code**				
**Any**	**39 (10.1%) 52**	**63 (17.0%) 89**	**102 (13.5%) 141**	**0.006**
**Infections and infestations**	**3 (0.8%) 3**	**5 (1.4%) 5**	**8 (1.1%) 8**	**0.50**
Bacteraemia	1 (0.3%) 1	0 (0.0%) 0	1 (0.1%) 1	
Lower respiratory tract infection	1 (0.3%) 1	2 (0.5%) 2	3 (0.4%) 3	
Pulmonary tuberculosis	0 (0.0%) 0	1 (0.3%) 1	1 (0.1%) 1	
Urinary tract infection	0 (0.0%) 0	1 (0.3%) 1	1 (0.1%) 1	
Respiratory tract infection	0 (0.0%) 0	1 (0.3%) 1	1 (0.1%) 1	
Spinal cord abscess	1 (0.3%) 1	0 (0.0%) 0	1 (0.1%) 1	
**Respiratory, thoracic and mediastinal disorders**	**2 (0.5%) 4**	**0 (0.0%) 0**	**2 (0.3%) 4**	**0.50**
Pneumonia aspiration	1 (0.3%) 2	0 (0.0%) 0	1 (0.1%) 2	
Respiratory failure	1 (0.3%) 2	0 (0.0%) 0	1 (0.1%) 2	
**Gastrointestinal disorders**	**8 (2.1%) 9**	**24 (6.5%) 32**	**32 (4.2%) 41**	**0.003**
Abdominal pain	0 (0.0%) 0	1 (0.3%) 1	1 (0.1%) 1	
Diarrhoea	1 (0.3%) 1	8 (2.2%) 8	9 (1.2%) 9	
Gastrointestinal disorder	0 (0.0%) 0	1 (0.3%) 1	1 (0.1%) 1	
Gastrointestinal haemorrhage	0 (0.0%) 0	1 (0.3%) 1	1 (0.1%) 1	
Nausea	2 (0.5%) 2	9 (2.4%) 9	11 (1.5%) 11	
Upper gastrointestinal haemorrhage	0 (0.0%) 0	1 (0.3%) 1	1 (0.1%) 1	
Vomiting	6 (1.5%) 6	10 (2.7%) 11	16 (2.1%) 17	
**Hepatobiliary disorders**	**0 (0.0%) 0**	**2 (0.5%) 2**	**2 (0.3%) 2**	**0.24**
Cholestasis	0 (0.0%) 0	1 (0.3%) 1	1 (0.1%) 1	
Jaundice	0 (0.0%) 0	1 (0.3%) 1	1 (0.1%) 1	
**Skin and subcutaneous tissue disorders**	**7 (1.8%) 9**	**8 (2.2%) 9**	**15 (2.0%) 18**	**0.80**
Blister	1 (0.3%) 1	1 (0.3%) 1	2 (0.3%) 2	
Petechiae	0 (0.0%) 0	1 (0.3%) 1	1 (0.1%) 1	
Rash	5 (1.3%) 7	4 (1.1%) 5	9 (1.2%) 12	
Rash macular	1 (0.3%) 1	1 (0.3%) 1	2 (0.3%) 2	
Rash pruritic	0 (0.0%) 0	1 (0.3%) 1	1 (0.1%) 1	
**Renal and urinary disorders**	**1 (0.3%) 2**	**8 (2.2%) 10**	**9 (1.2%) 12**	**0.02**
Nephritis	0 (0.0%) 0	1 (0.3%) 1	1 (0.1%) 1	
Renal impairment	1 (0.3%) 2	4 (1.1%) 5	5 (0.7%) 7	

	Placebo N=388	Rifampicin N=370	Total N=758	p*
Acute kidney injury	0 (0.0%) 0	3 (0.8%) 4	3 (0.4%) 4	
**General disorders and administration site conditions**	**4 (1.0%) 4**	**13 (3.5%) 13**	**17 (2.2%) 17**	**0.03**
Discomfort	0 (0.0%) 0	1 (0.3%) 1	1 (0.1%) 1	
Drug interaction	2 (0.5%) 2	8 (2.2%) 8	10 (1.3%) 10	
Malaise	0 (0.0%) 0	2 (0.5%) 2	2 (0.3%) 2	
Oedema	0 (0.0%) 0	1 (0.3%) 1	1 (0.1%) 1	
Pain	0 (0.0%) 0	1 (0.3%) 1	1 (0.1%) 1	
Pyrexia	2 (0.5%) 2	0 (0.0%) 0	2 (0.3%) 2	
**Investigations**	**12 (3.1%) 13**	**12 (3.2%) 14**	**24 (3.2%) 27**	**1.00**
Alanine aminotransferase increased	1 (0.3%) 1	2 (0.5%) 2	3 (0.4%) 3	
Blood bilirubin increased	0 (0.0%) 0	5 (1.4%) 5	5 (0.7%) 5	
Blood creatinine increased	1 (0.3%) 1	1 (0.3%) 1	2 (0.3%) 2	
Drug level increased	3 (0.8%) 3	1 (0.3%) 1	4 (0.5%) 4	
Liver function test abnormal	4 (1.0%) 4	1 (0.3%) 1	5 (0.7%) 5	
Blood alkaline phosphatase increased	1 (0.3%) 1	0 (0.0%) 0	1 (0.1%) 1	
Inflammatory marker increased	1 (0.3%) 1	0 (0.0%) 0	1 (0.1%) 1	
Liver function test increased	2 (0.5%) 2	2 (0.5%) 4	4 (0.5%) 6	
**Injury, poisoning and procedural complications**	**1 (0.3%) 1**	**0 (0.0%) 0**	**1 (0.1%) 1**	**1.00**
Foreign body aspiration	1 (0.3%) 1	0 (0.0%) 0	1 (0.1%) 1	
**Blood and lymphatic system disorders**	**1 (0.3%) 1**	**3 (0.8%) 3**	**4 (0.5%) 4**	**0.36**
Eosinophilia	0 (0.0%) 0	2 (0.5%) 2	2 (0.3%) 2	
Leukopenia	1 (0.3%) 1	0 (0.0%) 0	1 (0.1%) 1	
Neutropenia	0 (0.0%) 0	1 (0.3%) 1	1 (0.1%) 1	
**Metabolism and nutrition disorders**	**2 (0.5%) 3**	**0 (0.0%) 0**	**2 (0.3%) 3**	**0.50**
Fluid overload	1 (0.3%) 1	0 (0.0%) 0	1 (0.1%) 1	
Hyperkalaemia	1 (0.3%) 1	0 (0.0%) 0	1 (0.1%) 1	
Hypernatraemia	1 (0.3%) 1	0 (0.0%) 0	1 (0.1%) 1	
**Psychiatric disorders**	**1 (0.3%) 2**	**0 (0.0%) 0**	**1 (0.1%) 2**	**1.00**
Confusional state	1 (0.3%) 1	0 (0.0%) 0	1 (0.1%) 1	
Disorientation	1 (0.3%) 1	0 (0.0%) 0	1 (0.1%) 1	
**Nervous system disorders**	**1 (0.3%) 1**	**1 (0.3%) 1**	**2 (0.3%) 2**	**1.00**
Dizziness	1 (0.3%) 1	0 (0.0%) 0	1 (0.1%) 1	
Headache	0 (0.0%) 0	1 (0.3%) 1	1 (0.1%) 1	

Note: Table shows number of participants with one or more episode (% of participants) [number of episodes] (e.g., '2 (20.0%) [3],' would indicate a total of 3 episodes in 2 participants)
* Fisher’s exact test

Supplementary References

1. Fine JP, Gray RJ. A Proportional Hazards Model for the Subdistribution of a Competing Risk. Journal of the American Statistical Association 1999;94:496-509.
